# Connecting stakeholder priorities and desired environmental attributes for wetland restoration using ecosystem services and a heat map analysis for communications

**DOI:** 10.3389/fevo.2024.1290090

**Published:** 2024-03-27

**Authors:** Connie L. Hernandez, Leah M. Sharpe, Chloe A. Jackson, Matthew C. Harwell, Theodore H. DeWitt

**Affiliations:** 1 Oak Ridge Institute of Science and Education, Newport, OR, United States; 2 Gulf Ecosystem Measurement and Modeling Division, US Environmental Protection Agency (USEPA), Gulf Breeze, FL, United States; 3 Pacific Ecological Systems Division, US Environmental Protection Agency (USEPA), Newport, OR, United States

**Keywords:** ecosystem services, tidal wetlands, restoration, stakeholders, nature’s benefits, decision-making

## Abstract

Framing ecological restoration and monitoring goals from a human benefits perspective (i.e., ecosystem services) can help inform restoration planners, surrounding communities, and relevant stakeholders about the direct benefits they may obtain from a specific restoration project. We used a case study of tidal wetland restoration in the Tillamook River watershed in Oregon, USA, to demonstrate how to identify and integrate community stakeholders/beneficiaries and the environmental attributes they use to inform the design of and enhance environmental benefits from ecological restoration. Using the U.S. Environmental Protection Agency’s Final Ecosystem Goods and Services (FEGS) Scoping Tool, we quantify the types of ecosystem services of greatest common value to stakeholders/beneficiaries that lead to desired benefits that contribute to their well-being in the context of planned uses that can be incorporated into the restoration project. This case study identified priority stakeholders, beneficiaries, and environmental attributes of interest to inform restoration goal selection. This novel decision context application of the FEGS Scoping Tool also included an effort focused on how to communicate the connections between stakeholders, and the environmental attributes of greatest interest to them using heat maps.

## 1 Introduction

Ecosystem restoration is pursued for a variety of reasons, including improving the condition of the environment, and increasing the benefits for people who use, rely on, or care about the restored environment (ecosystem services; ES). Recognition of the need and efforts to consider and integrate ecosystem services into environmental decision-making has become an increasingly integral aspect of ecosystem-based management (Olander et al., 2018). As a result, funding for restoration projects increasingly incorporates design and justification for flows from the ecosystem to human well-being to leverage funding and stakeholder support (Jackson et al., 2022; Rossi et al., 2022).

A challenge with efforts to incorporate and quantify ES in restoration is that often, biophysical attributes of ecosystems are expressed as services without describing the full benefits to humans (DeWitt et al., 2020), or they measure economic values of end uses of ES (e.g., Russell et al., 2020). These approaches often leave out a large subset of possible ES that hold high cultural and social value (Chan et al., 2012). Studies have shown that when residents are asked to measure the value of nearby natural spaces, they highly value social and cultural benefits without attaching monetary values to those benefits (Pedersen et al., 2019).

Specifically, Final Ecosystem Goods and Services (FEGS) are those aspects of the environment that are directly enjoyed, used, or consumed by humans (Boyd and Banzhaf, 2007). They are specified as *final* because of the *direct* benefit they provide to humans (Boyd and Banzhaf, 2007; Landers and Nahlik, 2013; DeWitt et al., 2020). The consideration of FEGS can include a focus on those services that hold high cultural and social value.

With many constraints in ecological restorations, and with the expansive array of ES and benefits to humans, prioritizing which services to integrate into restoration can be challenging for managers. One approach to address these challenges is to conduct an analysis of ES priorities from related restoration projects locally, regionally, nationally, or by type of organization (e.g., Yee et al., 2019; Jackson et al., 2024) and develop a list of potential services of interest that can be considered for the local restoration context. This is especially useful when supporting literature is robust. Another approach is to determine what ES are of greatest interest to stakeholders and the general community (Chan et al., 2012; DeWitt et al., 2020; Sharpe et al., 2020).

The U.S. Environmental Protection Agency’s (EPA) National Ecosystem Services Classification System Plus (NESCS Plus; Newcomer-Johnson et al., 2020), uses three components to classify ES: 1) an environment type (i.e., where the service is produced); 2) the beneficiary or user (i.e., the role(s) people have when caring about the service); and 3) an ecological end product (i.e., the attribute of nature from which the benefit is derived). The FEGS Scoping tool, using NESCS Plus as its foundation, offers a way to identify priority interests for a given decision context (here, initial stages in restoration planning) in a transparent manner. This tool is designed to avoid conflicts by clearly articulating what stakeholder interests are, finding shared interests where they may or may not be obvious, and communicating what environmental attributes are relevant to support those interests (Sharpe et al., 2020). The FEGS Scoping tool uses a tiered multi-criteria decision analysis (MCDA) approach to highlight priorities of a decision. The tool uses MCDA to rank alternatives by the sum of weighted criteria. The tool’s objectives are to prioritize stakeholders, prioritize beneficiaries, and prioritize environmental attributes, with each step feeding into the next. For details about the tool, including the underlying methodology, the reader is referred to Sharpe et al. (2020) and Sharpe (2021).

This case study of a wetland restoration project in Tillamook Bay, Oregon is a demonstration of how to use an ES classification system that incorporates a wide array of environmental benefits (Newcomer-Johnson et al., 2020) and a tool that facilitates a systematic identification of stakeholder interests associated with specific environmental decision contexts (Sharpe et al., 2020). The objective of this study was to provide the Tillamook Estuaries Partnership (TEP) team with a structured approach for identifying stakeholder interests and incorporating those ES relevant to their interests into restoration goals. This was done by applying the FEGS Scoping tool to the final decisions on the restoration of the tidal wetland of the Tillamook river. While the results are specific for the Tillamook Bay restoration effort, the approaches and application of the FEGS Scoping Tool are applicable to other decision contexts.

### 1.1 Case study site

The Tillamook Bay basin, located along the coast of northern Oregon, is inextricably linked to the natural environment through fisheries, forestry, agriculture (particularly dairy farming), and nature-based recreation and tourism (Tillamook Estuary Partnership (TEP), 2019). Legacy forestry and farming practices degraded or transformed many of the wetlands in the watershed, and their loss has been associated with increased low-land flooding, reduced salmon populations, and degradation of water quality (Tillamook Estuary Partnership (TEP), 1999; Komar et al., 2004; Tillamook Estuary Partnership (TEP), 2019). Restoration of tidal wetlands is a priority of the Tillamook Estuaries Partnership, a National Estuaries Program site established in 1994, to improve water quality and wetlands habitats within Tillamook County (Tillamook Estuary Partnership (TEP), 1999, 2019).

**FIGURE 1:**
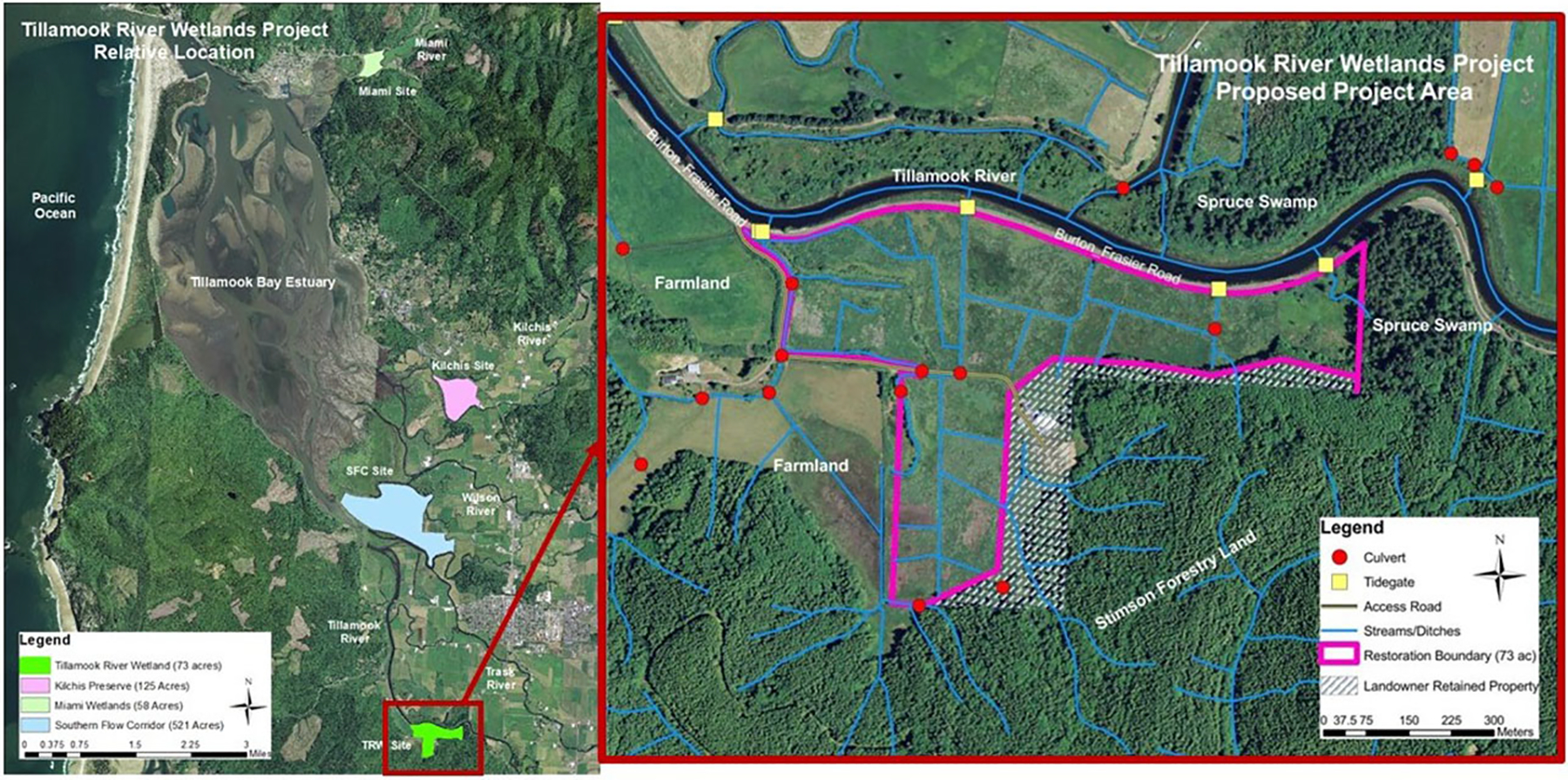
Tillamook Bay and several recent TEP restoration sites. The Tillamook River Wetlands restoration site, on the southern end of the bay is shown in more detail on the right, which shows the restoration boundary, streams and ditches, tide gates, and culverts. Outside the restoration site, the western side is mostly farmland, managed forest to the south, and spruce swamp to the east and northeast across the Tillamook River (Oregon Watershed Enhancement Board (OWEB), 2017).

The TEP is leading the restoration planning and implementation at a site called the Tillamook River Wetlands (TRW), a 73-acre site at mile three of the Tillamook River (Figure 1). The existing road infrastructure in the site experiences frequent flooding and thus often experiences damage and need for repairs. Historically, tidal wetlands around Tillamook, including within the TRW Restoration site, were significantly covered by Sitka Spruce (*Picea sitchensis*) swamp habitat (Brophy et al., 2019a). There was deforestation in the watersheds that feed into Tillamook Bay between 1931–1954 (Komar et al., 2004), including extensive loss of Sitka Spruce (Brophy, 2019). In the greater Tillamook Bay, an estimated 62.8% of tidal forest wetland and 63% of scrub shrub wetland still exists (Brophy, 2019). The TRW Restoration site has some forested wetland, specifically Sitka spruce remaining, a fraction of historical cover since the area was diked and drained (Oregon Watershed Enhancement Board, (OWEB) 2017). The site was acquired in 2020 by the North Coast Land Conservancy (NCLC) as a perpetual conservation easement and to “help protect and restore healthy watersheds and natural habitats that support thriving communities and strong economies.” (OWEB, n.d.). When acquired, the site consisted of palustrine emergent wetlands (86.6%), palustrine forested wetland (2.7%), and upland habitats (10.5%) (Oregon Watershed Enhancement Board, (OWEB) 2017). The tidal wetlands in Tillamook Bay are used by the federally threatened Oregon Coast Coho Salmon (*Oncorhynchus kisutch*) in addition to seven other salmonid species and 17 other known federally or state recognized species of concern (Oregon Watershed Enhancement Board, (OWEB) 2017). At the time of the study, the TRW partners were analyzing alternatives in the restoration design. The alternative analysis provided the county and private landowners with restoration alternatives that include consideration of climate change and infrastructure pressures (Tillamook Estuary Partnership (TEP), 2022).

For the TRW Restoration project, an increased trend in flooding events constitutes the major impetus for the need to consider restoration interventions. Currently, the site has a road that runs along the south bank of the Tillamook River, and several points of the road system flood 20–50 times a year. While supporting infrastructure has been installed (e.g., scour protection aprons between the road and river edge), those features are beginning to fail and there is significant deterioration of older road segments (Tillamook Estuary Partnership (TEP), 2022). Flood risk in Tillamook is increasing, and projections include increased erosion that could increase damages and costs to properties and structures (Komar et al., 2004; Oregon Department of Fish and Wildlife (ODFW), 2006; Brophy et al., 2019b). The TEP team recognized that there were multiple stakeholders for the TRW Restoration project with diverse benefits, and interests beyond the need to reduce and mitigate flooding.

The TRW Restoration managers and partners (Tillamook County Public Works, North Coast Land Conservancy, and Oregon Watershed Enhancement Board (OWEB)) are invested in incorporating stakeholder interests into project planning and implementation, and garnering project support from affected communities from the early planning stages. The TRW Restoration site, though rural, is surrounded by people with a variety of recreational and economic interests such as forestry, hunting, angling, farming, grazing, and boating (Gray, 2000). Studies have shown that coastal Oregon communities have experienced considerable demographic and economic changes. Overall, coastal areas have seen decreases in resource-based industries such as commercial fishing and timber, while personal incomes and employment associated with businesses serving tourism and retirees have increased (Gray, 2000; The Research Group, 2006; Ackerman et al., 2016). A study published in 2006 found that Tillamook still has a significant agricultural industry, 13.1% of total personal incomes, whereas tourism constitutes 3.8% of total personal incomes, lower than the coast-wide estimate of 5.6% of total incomes contributed by tourism. Oyster production in Tillamook Bay has decreased from an estimated 30,916 gallons in 1984 to 12,151 gallons in 2003 (The Research Group, 2006). Timber continues to be significant though the industry has been affected by a series of forest fires in Tillamook County from up until the 1950s (Gilden and Conway, 1999). An economic outlook by Gilden and Conway estimated that the timber would increase, and in 2006 was estimated to be 12.0% of Tillamook’s total personal incomes (Gilden and Conway, 1999; The Research Group, 2006). By 2019, outdoor recreation was determined to be a significant economic sector in Tillamook County, with over $737 million spent to support recreational activities and visitors (Mojica et al., 2021).

Past restoration efforts in other Tillamook Bay watersheds have included holding stakeholder hearings to formulate and vote on preferred restoration alternatives recognizing disparate viewpoints of potential outcomes (Gregory and Wellman, 2001). For example, the impetus of a large restoration project in an adjacent watershed that feeds into Tillamook Bay, the Southern Flow Corridor, was a storm that resulted in millions of dollars of damages in 2006. The Southern Flow Corridor restoration projects had points of public contention during planning, thus was forestalled and forced into a third-party mediation (Levesque, 2013; Haeffner and Hellman, 2020). While the project was successful at decreasing flooding in adjacent areas, the importance of other ES and benefits (e.g., diverse recreation and education benefits noted as important to stakeholders) (Janousek et al., 2021; Shaw and Dundas, 2021) was not measured.

At the outset, TEP and its partners were identifying alternatives to analyze for restoring the TRW Restoration site (Tillamook Estuary Partnership (TEP), 2022). For this project, we applied the tool considering that some of the restoration facets would likely need to include reconstructing the tide channel, and either upgrading or removing the road. In removing the road, a road outside the site would also be upgraded to withstand more traffic.

## 2 Methods

The TEP team was interested in having a structured approach for identifying stakeholder interests, and reconciling possible conflicts, while identifying those ES needed to sustain stakeholder interests and can be incorporated into restoration goals. The results of our analysis might then be used to inform the development of nominal restoration goals for this site and frame forthcoming discussions with local stakeholders This case study used the extensively peer-reviewed, publicly available FEGS Scoping Tool (Sharpe, 2021) in collaboration with TEP restoration managers, who were interested in how best to engage stakeholders in the planning process. Oftentimes, there are distinct stakeholders who have clearly competing interests, obscuring whatever shared interests they may have, so the TEP restoration managers wanted to use the FEGS Scoping Tool to help find shared interests among a possibly wide range of collective interests. The FEGS Scoping Tool was used to take a comprehensive and structured approach to identify stakeholders and the ways they are, or could be, benefiting from the area being considered for restoration. The tool required managers to take a more deliberative approach towards considering stakeholders and benefits. This allowed managers to identify groups that might otherwise be overlooked, capture potential impacts that managers should be prepared to address during discussions with stakeholders, and identify groups that may not be aware that this effort will affect them.

We worked with the TEP restoration managers, using the steps and guidance of the FEGS Scoping Tool (see Sharpe et al., 2020 and Sharpe, 2021 for tool methodology), to define the decision context; to identify and prioritize stakeholders; to define the benefits that stakeholders were interested (beneficiaries); and to determine the environmental attributes for each beneficiary. The tool requires each criterion be scored for the decisions at hand, and that each stakeholder group be identified and evaluated toward each criterion. Secondly, the environmental benefits of each stakeholder group need to be identified. And third the environmental attributes needed to realize each type of benefit must be identified. Each step is guided by well-defined choices specified in lists within the software. Thus, the tool requires many data inputs, which were determined through interviews of two TEP restoration managers conducted by a team of four EPA researchers. This data input process was accomplished as a series of virtual meetings to discuss the project and walk through the tool. The FEGS Scoping Tool was used very early in the decision-making process to help inform the upcoming evaluation of engineering alternatives that could be made to existing tide gates, roads, and other infrastructure.

**FIGURE 2:**
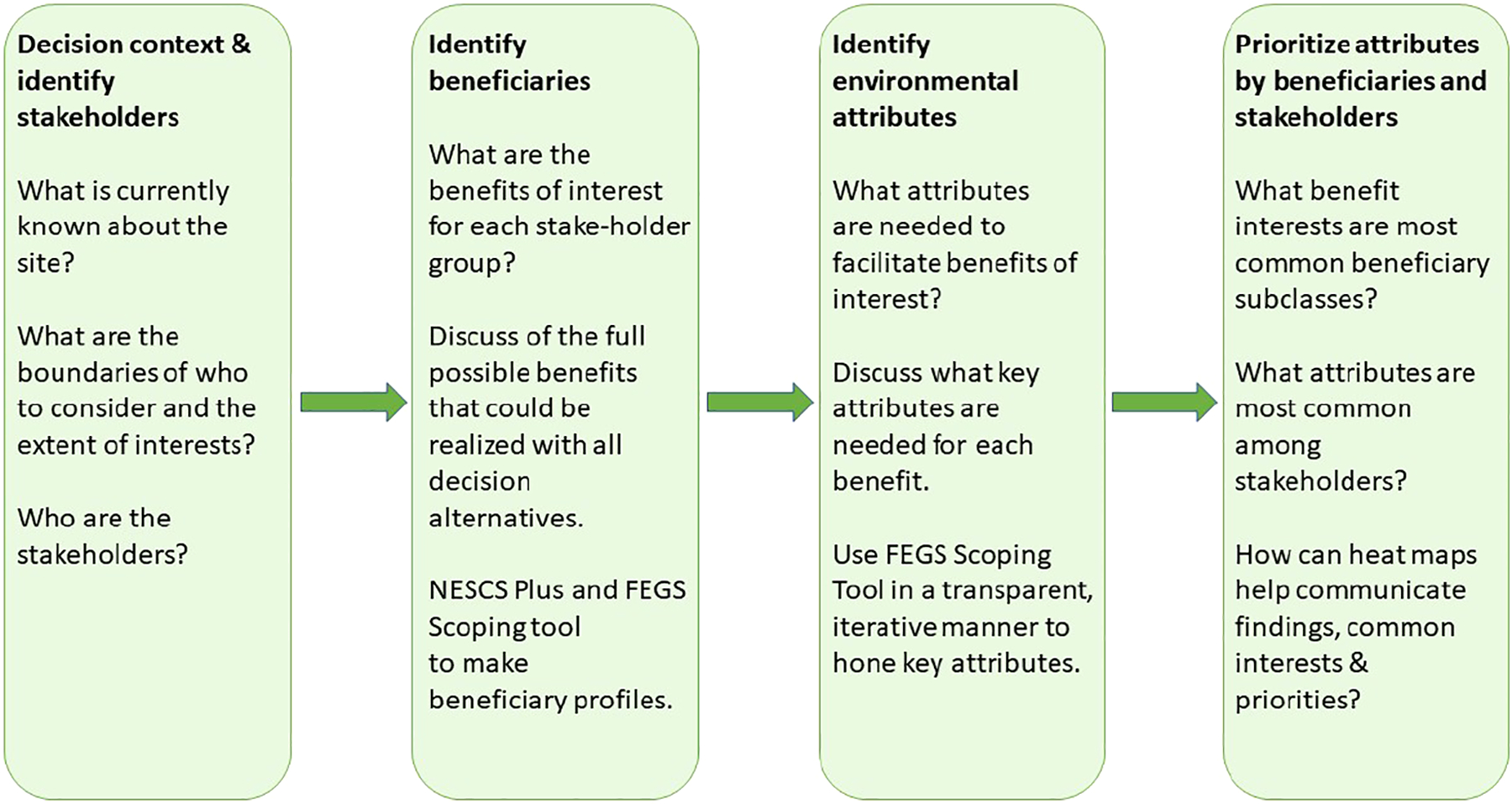
Conceptual diagram of the steps used to apply the FEGS Scoping Tool and other analyses for the Tillamook River Wetlands Restoration site.

The FEGS Scoping Tool uses a tiered approach to MCDA using key criteria, explicitly evaluated by the people making the decision or using the tool, to prioritize stakeholder groups interests when evaluating alternatives and with limited resources (Sharpe, 2021). The TEP restoration managers requested a visual-communication tool output that could more explicitly connect the environmental attribute results back to individual stakeholder groups, and to individual beneficiary groups (Figure 2; Hernandez et al., 2022). The tool produces bar charts that indicate the relative prioritization for each of the stakeholders, beneficiaries, and environmental attributes (Sharpe, 2021). Furthermore, we used the calculations built in the FEGS Scoping Tool software to do a crosswalk between the environmental attributes and the suite of stakeholder groups as identified by the TEP restoration managers. To do this, we populated the raw data from the tool into a simple spreadsheet, color-coding cells different shades depending on the value of a given cell relative to the overall range of results to develop a heat map for visually displaying results. Each step of using the FEGS Scoping Tool for this case study is described in the following four sections.

### 2.1 Decision context, identifying stakeholders

In the stakeholder prioritization step, the key decision criteria that the tool asks users to consider are: interest; influence; impact; urgency; proximity; economic interest; rights; fairness; and underrepresented/underserved populations (Sharpe et al., 2021). The decision context for this prioritization was clarified through discussions with the TEP restoration managers of what the impetus was for the land acquisition, the current knowledge of pressing hydrological issues at the site, the impacts to surrounding communities, and both current and likely stakeholder interests in connection to the site and restoration decisions and outcomes. Setting the decision context included defining the geographic bounds of adjacent areas and identifying stakeholders that would potentially be impacted by: 1) being near the restoration site boundaries; and 2) the uses associated with road access through the site given that a nearby road could be modified to replace the current road if the current road running through the site is removed.

The stakeholder prioritization criteria are weighted by the tool users or decision makers to transparently convey which criteria matter most to those using the tool and/or making the decisions when determining the relative priority of the stakeholder groups. Subsequently, each stakeholder is scored on those criteria according to what degree the stakeholder group met each criterion. The values recorded in these two initial steps propagate through the FEGS Scoping Tool analysis as the prioritized stakeholder groups affect the prioritization of beneficiaries and of the environmental attributes associated with the restoration of the TRW Restoration site (Sharpe et al., 2020; Sharpe, 2021).

The TEP restoration managers had completed some initial background information collection and restoration planning activities for site and surrounding areas, such as communicating with some key stakeholders before beginning the process of the FEGS Scoping Tool application. Their knowledge was instrumental in being able to identify and characterize the most likely stakeholder groups to consider and the benefits those stakeholders were seeking from the restoration site. In total, 15 stakeholder groups were identified (Table 1; Hernandez et al., 2022). Initial conversational meetings were held to discuss the site’s current conditions, identifying stakeholder groups, and weighting the decision criteria from the perspective of the restoration managers. The restoration managers identified how each of the stakeholder groups met each of the decision criteria.

### 2.2 Identify beneficiaries

**FIGURE 3:**
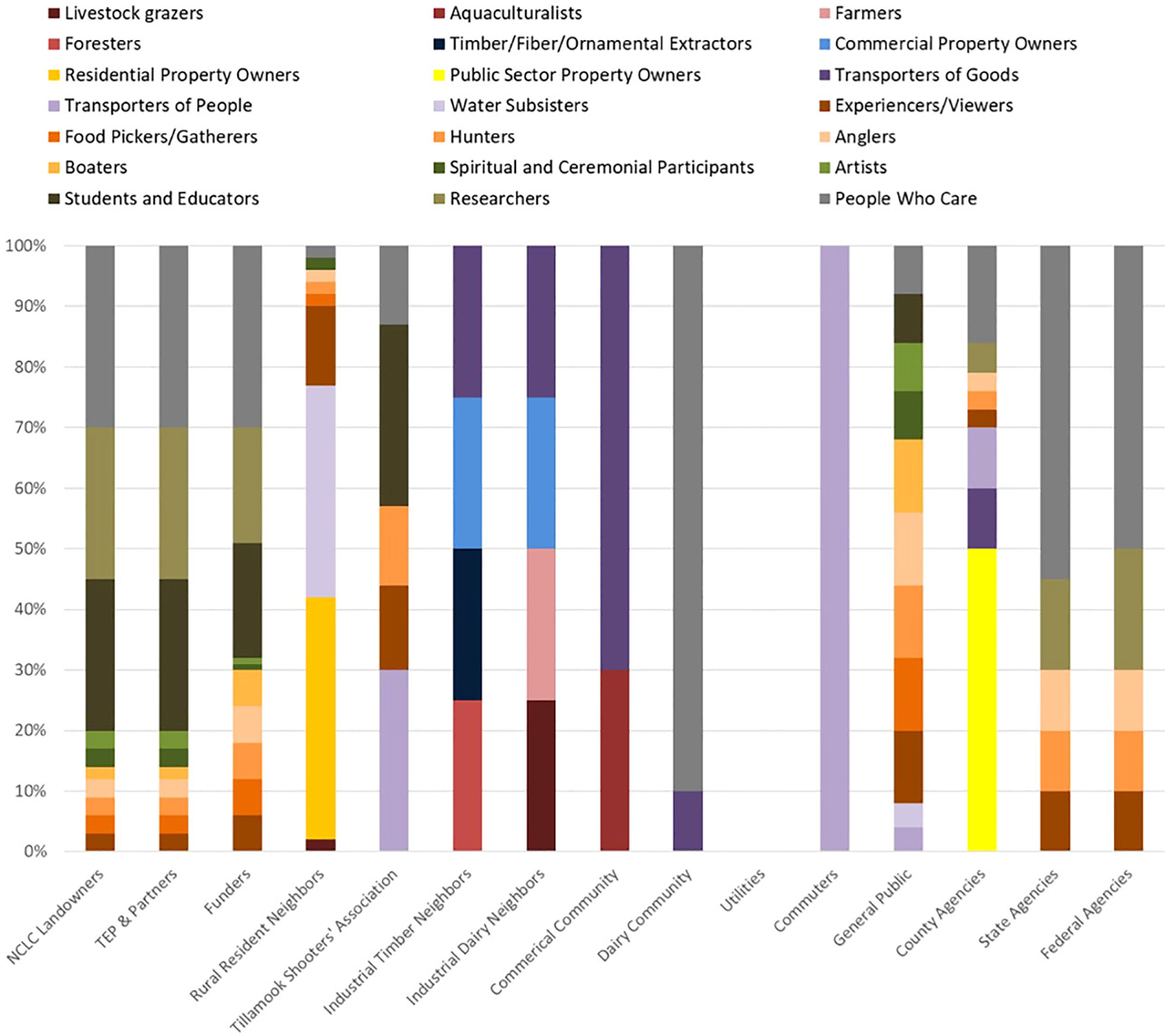
Beneficiary distribution for each stakeholder group; Utilities (local utility companies) were a stakeholder group that was considered to likely have no beneficiary interests at the site. (Reproduced from Hernandez et al., 2022).

After stakeholder groups are identified and prioritized according to the decision context criteria, the FEGS Scoping Tool asks the user to characterize how each stakeholder group benefits from nature (i.e., identifying the beneficiary profile) according to the beneficiary classification defined in the NESCS Plus (Newcomer-Johnson et al., 2020). Each stakeholder group was segmented, by percentage, into the ways they benefit from nature (Figure 3).

Keeping in mind the ecological setting of the site, the decision context boundaries, geographic boundaries, and the interests of stakeholder groups, a beneficiary profile for each group was created to better understand the ways in which a group may be impacted as a result of changes to the TRW Restoration site. The ways in which each group interacts with the ecological setting and geographic boundary informs what nature-based benefits may already be produced by the site or are of interest in being addressed through the restoration process. Some benefits are used or enjoyed primarily at the site (e.g., viewing wildlife or minimizing flood damage) whereas other benefits are realized over an area larger than the site (i.e., vistas of wetland habitat, production of game fish) (Ringold et al., 2013). This variability in location where ES are enjoyed relative to where they are produced affects the range of types of beneficiaries that will be affected by restoration of the TRW Restoration site.

### 2.3 Identify environmental attributes

The last step regarding the inputs in the FEGS Scoping Tool was to identify the environmental attributes necessary for each beneficiary to receive the benefits they value in the context of the decision and location of the site. The environmental attribute categories and subcategories in the FEGS Scoping Tool guidance follow the suite of categories and definitions from the NESCS Plus (Newcomer-Johnson et al., 2020).

The environmental attributes step was approached by identifying what individual attributes are needed to sustain the interests and uses of the beneficiaries at the site for the decision context. Some benefits may be used or enjoyed within a site’s boundaries (e.g., wildlife viewing, extraction of timber) whereas other benefits are produced over an area larger than the site (e.g., vistas of wetland habitat, production of game fish) (Ringold et al., 2013). While a beneficiary may care about multiple attributes of the environment within a restoration site, when assuming a specific beneficiary role, we made a concerted effort to consider the most relevant biophysical attributes (such as flooding and water quality) needed to directly use, consume, or appreciate the environment in order to fulfill the specific benefit for the beneficiary role.

### 2.4 Prioritize beneficiaries and attributes

The FEGS Scoping Tool uses a tiered version of the multi-criteria decision approach, known as ranking the alternatives, on the sum of weighted criteria (Sharpe et al., 2020; Sharpe, 2021). In the first step, the decision criteria are weighted and used to score the stakeholder groups. The combination of weighting and scores results in a prioritization value for each group. This result is then used as the weight in the second step. The result of the second step, the prioritization value of each beneficiary group, is used as the weight in the third step. This means that, in the raw data of the weights and scores, it is possible to show the relative priority of the attributes for each stakeholder group, even though it is not an explicit tool output. This analysis allowed managers to see common attributes of interest across stakeholder groups explicitly, in addition to the tool output display of common attributes of interest across beneficiary groups. This was done externally to the tool itself, using the same inputs as the tool.

## 3 Results

Given that the FEGS Scoping Tool focuses on information to help evaluate: (1) what benefits stakeholder groups are interested in for the site; and (2) what environmental attributes are needed to realize those benefits, results below include both qualitative and quantitative information. It is important to note that this study primarily focuses on the human-dimension elements of the early phases of planning a wetland restoration project, and the TEP restoration managers were interested in both qualitative and quantitative outcomes of this study.

### 3.1 Decision context

The decision context was set based on the restoration managers’ knowledge at the time of discussing and recording the FEGS Scoping Tool inputs, and their expected next steps in the restoration planning process. The criteria do not have to be considered independently of one another. For the TRW Restoration project, the most important criteria were level of influence and rights, each of which were equally weighted. In total, eight criteria had scores greater than 50 as determined by the restoration managers (Table 2). The decision makers or tool users (in this case, the users were the restoration managers, with discussion with EPA researchers) determine which of the decision criteria are most meaningful to them when distinguishing among priorities and the stakeholder groups (Sharpe et al., 2020, 2021; Hernandez et al., 2022). Though the criteria reflect the values set by the tool users, the tool makes this step transparent for valuing the criteria that are key to stakeholder and decision analyses (Belton and Stewart, 2002; Gregory et al., 2012; Sharpe et al., 2021).

When considering the influence criterion and establishing weights, the TEP restoration managers felt that the authority to approve or strike down restoration design and interventions was meaningful for distinguishing among stakeholder groups. Some stakeholders may have significant informal influence on other stakeholders, thereby affecting decisions about the design and implementation of the project. The TEP restoration managers felt those groups with the ability to block or significantly influence plans should be prioritized. The critical effect that an authority could have on the project itself led to weighting influence as one of the most important criteria.

Although TEP restoration managers initially assigned 100% to the importance of rights (Table 2), groups that have property, legal, property, or formal user rights in the decision making and outcome of the restoration needed to be distinguished and given higher weight than through other criteria. It is important to note that the FEGS Scoping Tool can be used in an iterative fashion, allowing managers the opportunity to examine different weighing overall. Some stakeholders have the authority or legal standing to approve or block the restoration design or implementation. The first alternative may include major structural improvements to Burton-Fraser Road, which would create a detour north through Tillamook or south through Eckloff road, which would cause significant traffic delays for a minimum of two years during construction. The second alternative includes removing a portion of the current Burton-Fraser Road and upgrading Eckloff road, which would cause traffic delays while Eckloff road is under construction but would later cause minimal impact to traffic. However, current conditions of the Burton-Fraser Road are deteriorating bank protections, and portions of the road frequently flood during very high tides, naturally delaying commuter traffic (Tillamook Estuary Partnership (TEP), 2022). Both could potentially impact several stakeholder groups. Thus, impact was weighted highly (90%; Table 2) to elevate the importance of the impact to stakeholders who frequent roads in the area.

The TEP restoration managers wanted to consider how people who are nearest to the site will be affected by modifications to Burton-Fraser Road. They were also concerned about how restoration and future uses of the TRW property may impact surrounding property values, businesses, etc. Hence, the criterion for proximity weighted highly and at the same weight as magnitude and probability of impact.

There was no expected significant direct economic impact from the possible removal of a section of Burton-Fraser Road adjacent to the TRW Restoration site. An adjacent farm would be most affected by the road change, but the expected impact would be small. A road closure would cause commuters and tourists to take a slightly longer route, but the county would be relieved of the expense to frequently maintain and repair the road. Modifications to the land use might affect neighboring property values or land uses, although whether the likely net effect of TRW Restoration would result in an increase or decrease property value has not been determined, though the Southern Flow Corridor restoration resulted in a near term, net increase in surrounding property values (National Oceanic and Atmospheric Administration (NOAA), 2021). Economic interest was seen as a criterion that would be less impactful to decision making than the higher prioritized criteria.

The need to decide or implement changes within a certain timeframe varies from stakeholder to stakeholder, and managers were willing to consider time constraint needs under the urgency criterion. There were already existing expectations for when the decision should be made based on the availability of funding. There were additional temporal considerations based on the poor condition of the Burton-Fraser Road; costs to Tillamook County to repair the road could be avoided if an early decision were made to allow the TRW Restoration project to remove or modify the road.

Interest from the public is sought but will have less influence on the restoration design and plan approval decisions than other criteria. The TEP managers want to consider the expressed interests from all stakeholders, but other factors such as influence, rights, impact, and proximity were given greater weight as decision criteria.

Decision makers are likely to consider economic and property rights more heavily than fairness. The TEP restoration managers want to make sure that stakeholders do not feel left out of the process but consider that other criteria are more persuasive in the decision-making process.

Using EPA’s environmental justice screening and mapping tool EJSCREEN (United States Environmental Protection Agency (U.S. EPA), 2023) and a 2.0-mile ring centered at the TRW Restoration site, EPA researchers determined that no significant underrepresented or underserved communities may exist in proximity to the TRW Restoration site. A 2.0-mile ring centered at the TRW Restoration site has an approximate population of 4,395 people, which includes large parts of the town of Tillamook, and has a 45% rate of low-income population, which is a higher rate as compared to state and national averages (29% in Oregon, 30% average in the USA). All other EJSCREEN indicators for environment, demographics, and environmental justice within this radius were comparable to or below state and national averages. Thus, consideration of the concerns for underrepresented and underserved populations was given a low weight. If this assessment is incorrect, this criterion could be given greater weight and the analysis repeated. However, there may be other more nuanced reasons to determine whether communities impacted by the restoration project are environmental justice communities that cannot be captured due to EJSCREEN’s limitations.

### 3.2 Stakeholder prioritization

**FIGURE 4:**
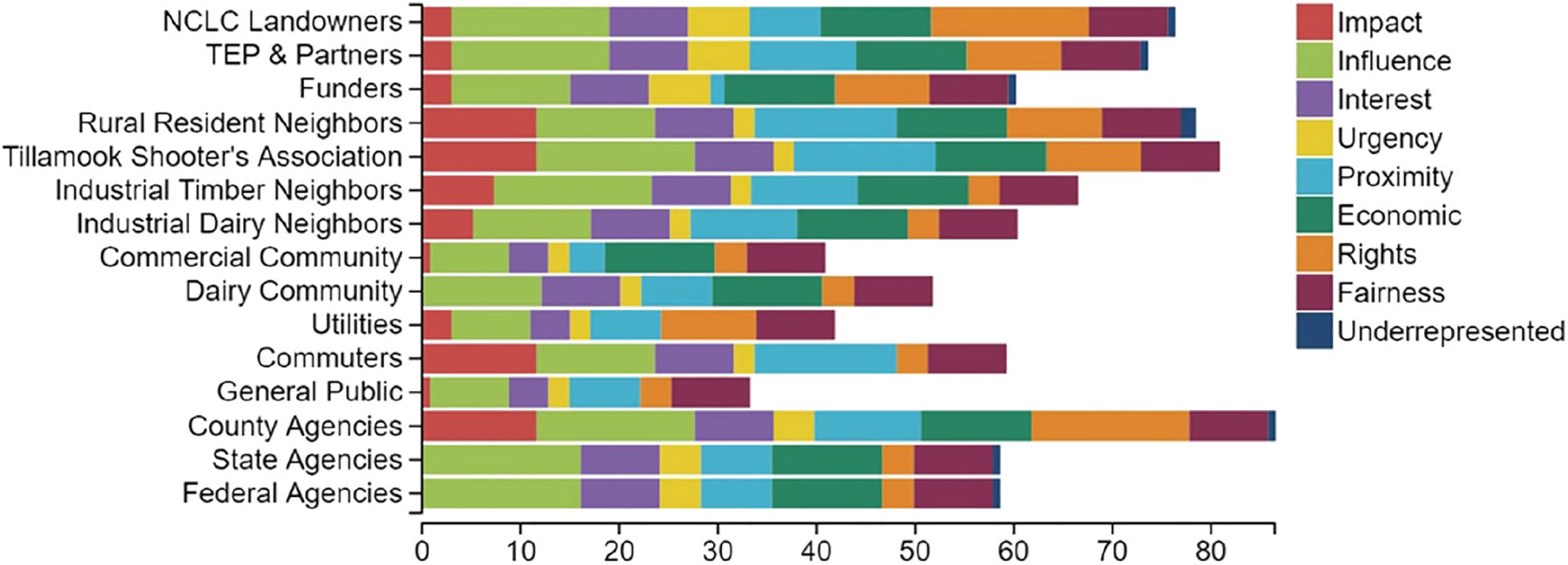
Relative priority of stakeholders based on their scores for each weighted decision criterion.

The results of the stakeholder prioritization can help show how stakeholders might be unexpectedly similar or disparate in how they fulfill any of the decision criteria, and how differing scores or emphasis placed by setting the decision context will affect the outcome of stakeholder prioritization. The Tillamook Shooters Association and County Agencies stakeholder might seem like disparate groups in how they fit the decision criteria, yet the two have similar scores across most decision criteria, except urgency, proximity, rights, and underrepresented and underserved populations. The TEP and partners, and NCLC Landowners are two stakeholder groups that have similar characteristics regarding the decision criteria, but they differ in proximity and rights. All stakeholder groups fulfilled, to some degree, the influence, interest, urgency, proximity, rights, and fairness criteria. All groups have scores of 100 for fairness, which was set because the restoration managers felt that every stakeholders’ interests need to be considered and might feel that each would say their interests need to be considered fairly in the decision-making process. State Agencies, Federal Agencies, and the Dairy Community stakeholders did not score for impact, and Utilities, Commuters, and General Public did not score for economic interest (Figure 4). The tool-generated stakeholder prioritization shows that Industrial Dairy Neighbors, Funders, Commuters, State Agencies, and Federal Agencies all have similar relative priority (Figure 4), though they have different contributing resulting priority for individual criterion, such as for impact, proximity, economic interest, or rights.

### 3.3 Beneficiary prioritization

**FIGURE 5:**
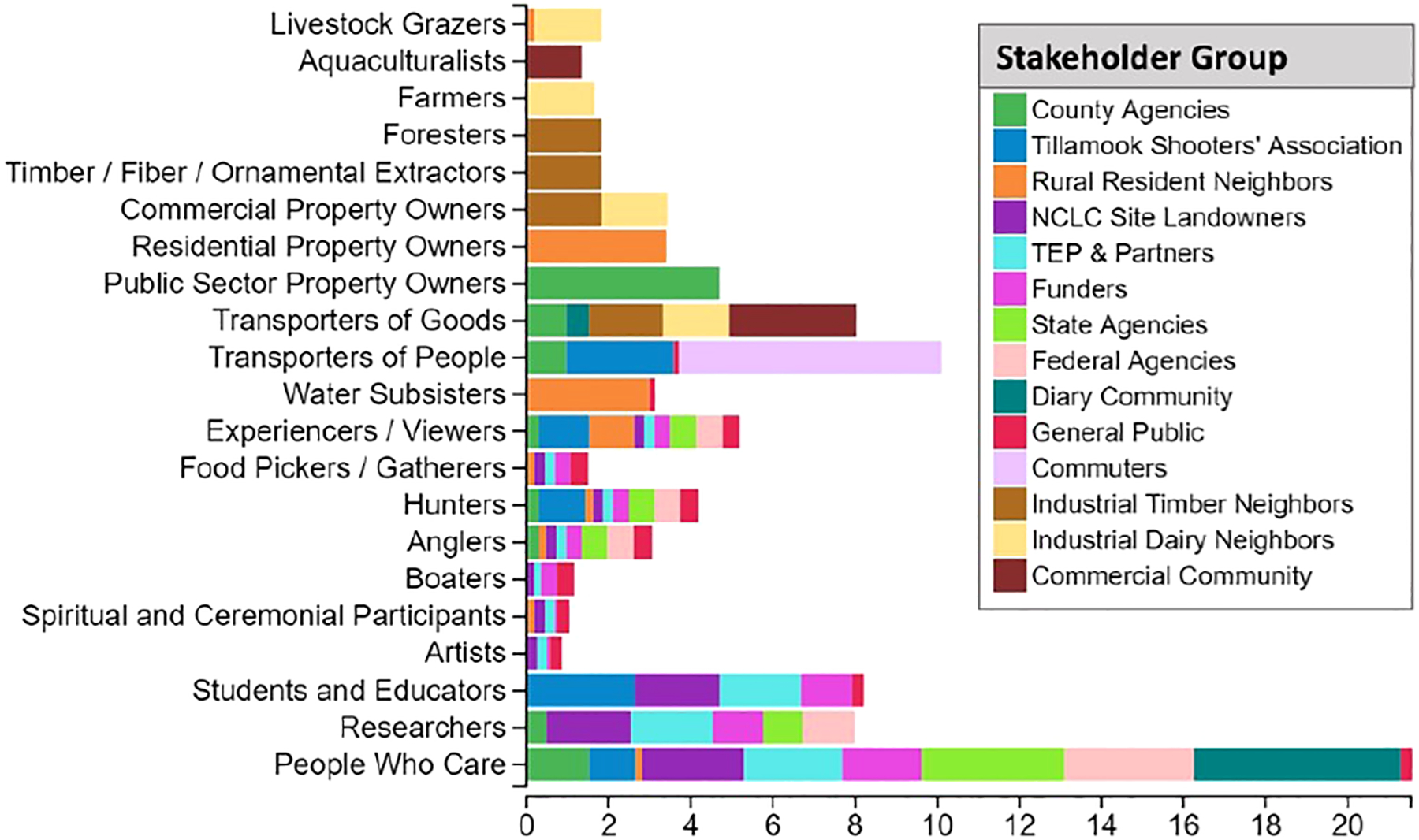
Relative priority of beneficiaries affected by Tillamook River Wetlands Restoration, determined by the beneficiary roles of each stakeholder.

The results from the beneficiary prioritization step of the tool shows the suite of beneficiary groups that the TEP restoration managers expect to be represented by stakeholder groups (Figure 5). A total of 21 beneficiaries were identified amongst all stakeholder groups. Of those, 12 beneficiary groups resulted in a relative priority value of 3.0 or above (Hernandez et al., 2022). Lower-scoring beneficiaries are likely to have less influence in the final prioritization of ecosystem attributes, yet their attribute interests could align with more highly ranked and thus influential beneficiary groups. The result can help decision makers generalize which beneficiary groups may have greater or lesser interest or potential to be generally impacted in by the restoration decision.

The beneficiary prioritization results help to show where stakeholders have shared interests (Figure 5). For example, the Tillamook Shooters Association is interested in youth education pertaining to safe hunting practices on adjacent property, so their beneficiary profile includes recreational hunters who are interested in potential production of game animals that can be bolstered by restoration decisions and then migrate onto the adjacent association’s property. The General Public group includes students and educators who will have access to the restored site and can use the area to learn about wetland ecology, processes, or associated species. Rural Residential Neighbors includes homeowners and renters who the TEP restoration managers felt will likely appreciate viewing local wetland plant communities. This identification of beneficiaries helps create a more comprehensive view of how different groups of people interact with the environment and creates opportunities to identify what uses or benefits are shared among stakeholders, especially when those shared interests may not be otherwise obvious (Sharpe et al., 2020).

The top beneficiary is People Who Care, a beneficiary role represented by ten stakeholder groups. However, the end points of what existence values people care about deserves to be more nuanced. The Dairy Community group includes people who care that ecosystems support farm production, which might be different than, or in addition to caring that tidal wetland ecosystems sustain healthy habitats for salmonid species. Many of the stakeholder groups that make up this beneficiary group were identified as caring that the TRW Restoration site be restored to tidal wetlands.

The second prioritized beneficiary role is Transporters of People. This beneficiary is highly prioritized because the Commuters stakeholder group only includes transporters of people as a beneficiary role. The Tillamook Shooters Association has 30% of its beneficiary profile for the transporters of people beneficiary. The General Public and County Agency stakeholder groups also have a role as Transporters of People.

Students and Educators were present as beneficiaries within the Tillamook Shooters Association, NCLC Landowners, TEP & Partners, Funders, and General Public stakeholder groups (Figure 5). There was interest by all these stakeholders to create opportunities for environmental education at the site and to educate the public about ecological and wildlife features at this site.

Transporters of Goods was included in the beneficiary profiles of industrial/commercial stakeholders (Commercial Community, Industrial Timber, Industrial Dairy Neighbors, and greater Dairy Community; Figure 5), who use the stretch of Burton-Fraser Road adjacent to the site for transporting their goods. This is especially true for commercial stakeholders located close to the TRW Restoration site.

The NCLC landowner, TEP & Partners, Funders, State Agencies, and Federal Agencies included Researchers in their beneficiary profiles (Figure 5). All these stakeholders are interested in conducting or supporting environmental research at (or including) the TRW Restoration site. This includes research on tidal wetland restoration.

The Experiencers/Viewers beneficiary was included in the profiles of nine stakeholder groups (County, State and Federal Agencies, Funders, NCLC, TEP & Partners, Tillamook Shooters Association, Rural Resident Neighbors, and General Public). While a less tangible benefit, and often a very subjective one, a popular recreational draw in Tillamook Bay and the Oregon coast is the composite features of nature that are regarded as aesthetically pleasing. Oregon Department of Fish and Wildlife Marine Resources Program’s human dimensions research has surveyed visitors to the Oregon coast and found that going to the beach, sightseeing and wildlife viewing were the top two main activities and purposes for visiting the coast (Fox et al., 2022). Opportunities and access for outdoor experiences and views may serve alongside a diverse set of other activities that these stakeholder groups are interested in benefitting from and sustaining.

Public Sector Property Owners were only associated with County Agencies, but it comprised 75% of that influential stakeholder’s beneficiary profile. The county owns Burton-Fraser Road which floods frequently and is in need of repair. Modification or removal of the road were major considerations in the TRW Restoration design decisions.

Hunters were included in the beneficiary profiles of nine stakeholder groups (County, State, and Federal Agencies; Funders, General Public, NCLC Landowners, Rural Resident Neighbors, TEP & Partners, and Tillamook Shooters Association). State and federal agencies regulate hunting and have interest in maintaining recreational benefits and resources for hunters. The County Sherriff is interested in maintaining hunter safety. The TEP & Partners and NCLC have interest in creating and managing habitats for wildlife used by recreational hunters.

Industrial Dairy and Industrial Timber Neighbors include Commercial Property Owners as beneficiaries. These agricultural businesses rely on properties that are upland of the restoration site. Residential Property Owners was a benefit only associated with Rural Resident Neighbors, but it comprised 40% of that stakeholder’s beneficiary roles. While few residents comprise this group, they may have an outsized influence on the restoration plan which could potentially impact property values.

The General Public and Rural Resident Neighbors were the only stakeholder groups that included Water Subsisters as beneficiaries. As much as 96% of the score for this beneficiary was contributed by Rural Resident Neighbors.

Anglers were included in the beneficiary profiles of eight stakeholder groups (County Agencies, Rural Resident Neighbors, NCLC landowners, TEP & Partners, Funders, General Public, State, and Federal Agencies). State agencies permit and regulate fishing. All groups have interest in maintaining recreational benefits and resources for anglers, and the habitats of the species targeted by recreational anglers.

### 3.4 Environmental attributes

**FIGURE 6:**
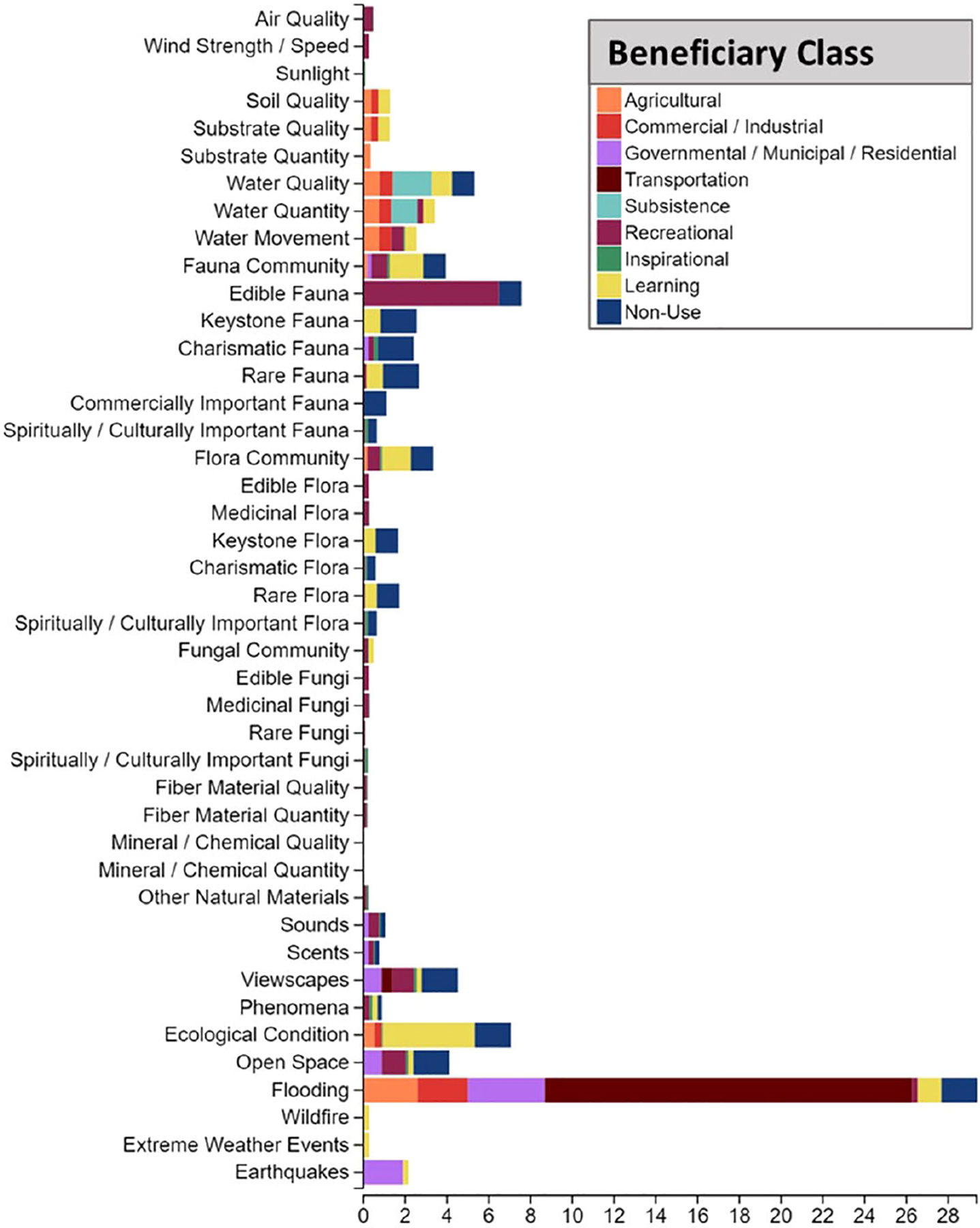
Relative prioritization of environmental attributes results from building a profile of suite of attributes that each beneficiary group cares about or needs. Beneficiary groups belonging to the same NESCS plus beneficiary class (e.g., Livestock Grazers, Aquaculturalists, Farmers, and Foresters collectively belong to the Agricultural beneficiary class) are grouped together in the legend.

The FEGS Scoping Tool results of environmental attributes shows which attributes of nature are important to each beneficiary group based on the NESCS Plus broader categorization of beneficiaries (e.g., Transportation includes the Transporters of Goods and Transporters of People beneficiaries; Newcomer-Johnson et al., 2020). There were 43 environmental attributes identified as part of at least one beneficiary’s profile (Figure 6).

Individuals may care about multiple aspects of the environment at a site but when acting as a specific beneficiary,there is a subset of biophysical attributes that provides the benefits that are necessary to provide the direct interests in using, consuming, or appreciating nature (i.e., valued environmental attributes). The number of environmental attributes valued varied among beneficiaries. Each beneficiary had 100 points to distribute across all attributes of interest or concern. Some beneficiary groups, such as Hunters, primarily valued edible fauna (and thus give this attribute a high score), while Students and Educators valued multiple subcategories of environmental attributes for the purpose of studying various components while visiting the site, meaning that there might be many attributes with relatively smaller results due to wider dispersed interests contributed by Students and Educators.

Highly prioritized environmental attributes may become more focal in driving decision making, setting goals for outcomes and monitoring. The top environmental attribute was flooding, which was valued by seven beneficiary groups and received the greatest contribution by Transporters of Goods and People, but it was also important to six additional broader categories of beneficiaries. This attribute reflects the composite natural features that mitigate flooding at the site, which was one of the driving concerns and impetus for the restoration easement. Edible fauna was the second most highly ranked environmental attribute, valued by Hunters, Anglers, and People Who Care. Edible fauna, like flooding, was highly prioritized in part because Hunters and Anglers have over 90% of their collective scoring going to edible fauna.

Ecological condition was a composite attribute that includes the overall ecosystem(s) and the associated physical, chemical, and biological processes, communities, and characteristics. It was one of the top attributes because most beneficiaries included this attribute in their profile. In discussions between the researchers and TEP restoration managers, we decided to include various composite interests related to ecological condition, but only those associated with other endpoints such as the holistic environmental conditions needed for supporting farming, artistic and inspirational uses, outdoor learning, and research events.

Water quality was an attribute important to agricultural beneficiaries, which include Aquaculturalists, Farmers, and Livestock Grazers who mainly use adjacent land parcels. About half of the overall results for this attribute also came from Non-Use (People Who Care) and Subsistence beneficiaries. Different aspects of water quality conditions include endpoints for farming, downstream oyster farms, and private well water use, in addition to the importance some might place to simply know that the water quality meets certain desirable criteria. Other beneficiaries who place an importance on water quality included Learning beneficiaries (Researchers, Students and Educators) who may use the site to study or learn about water properties and related biophysical processes. It may be important to note that specific properties and parameter thresholds to characterize water quality may be different for different beneficiaries and stakeholders.

Viewscapes was an important attribute to Non-Use beneficiaries (People Who Care), and Recreational (Experiencers and Viewers), Inspirational (Artists, and Spiritual and Ceremonial Participants) and Residential beneficiaries. Residents may be motivated to live in the area for the view from their properties, Transporters of People as drive-by-sightseers, and Students and Educators for both the view and accessibility of the site as an outdoor classroom. Seven total beneficiary subclasses cared for this composite attribute.

Open space was a composite important attribute to various beneficiaries in Learning, Inspirational, Recreational, Non-Use, and Government/Municipal/Residential classes. The NESCS Plus refers to open space as an opportunity for urban development (Newcomer-Johnson et al., 2020), but because the TRW Restoration site was designated for conservation, we used this attribute to refer to the long-term existence of undeveloped, green open space.

Fauna community was an important attribute to seven beneficiaries: Aquaculturalists, Residential Property Owners, Experiencers/Viewers, Artists, Students and Educators, Researchers, and People Who Care. For this attribute, the specific benefits for an individual beneficiary group were expected to vary. The fauna community that benefits Aquaculturalists may be different than the fauna community that draws in artists or researchers.

Water quantity was important to Water Subsisters, Researchers, Livestock Grazers, Aquaculturalists, Farmers, Experiencers/Viewers, Boaters, and Commercial Property Owners. The largest portion of the results was contributed by Water Subsisters, who are made up of the neighbors that depend on private well systems. The specific benefits of this attribute included home use, water for livestock and forestry, and small craft navigation.

Flora community was valued by Aquaculturalists, Artists, Experiencers/Viewers, Food Pickers/Gatherers, People Who Care, Students and Educators, and Researchers. The specific benefits of this attribute for an individual beneficiary also may vary, as the flora composition that benefits Aquaculturalists may be different than what attracts Artists or for People Who Care that a diverse, native vegetation community exists at the site.

### 3.5 Beneficiaries x environmental attributes

The TEP restoration managers were interested in exploring how beneficiary subclasses contribute to environmental attribute results, and how environmental attributes are distributed among stakeholder groups. The tool uses an MCDA approach to rank the alternatives on the sum of weighted criteria (Sharpe et al., 2020). We used the data from the weights and scores to analyze the relative priority of environmental attributes for each beneficiary subclass (Table 3), and the environmental attributes for each stakeholder groups (see section below; Table 4).

The tool produces the environmental attribute prioritization result based on the broader categorization of beneficiaries by NESCS Plus (Figure 6; Newcomer-Johnson et al., 2020). While the FEGS Scoping Tool results make the visual representation more straightforward to convey, it can be challenging to attempt to tease out how much an individual-beneficiary contributions to environmental attribute results. We used the spreadsheet calculations of the FEGS Scoping Tool data outputs to explore the attribute prioritization result at a finer scale and create a heat map to examine the distribution of attribute scores for each beneficiary group (Table 4).

The recreational class of beneficiaries has five subclasses that include Anglers, Food Pickers/Gatherers, and Hunters. The results of the heat map on Table 3 show that the edible fauna attribute is highly valued by Hunters and Anglers, but not by Food Pickers/Gatherers, suggesting that specific types of edible fauna and degree of importance can vary among beneficiary subclasses of the same class. The learning class of beneficiaries is made up of Students and Educators and Researchers, and ecological condition is significantly valued by Students and Educators for the interpretation that educational opportunities at the restoration site may focus on an overview of composite ecological condition.

### 3.6 Stakeholders x environmental attributes

The restoration managers were also interested in exploring how the environmental attribute results related to the stakeholder groups, which is not an output provided by the FEGS Scoping Tool. The top nine environmental attributes that had a total result score of 3.00 or higher are represented in Table 4 (see Supplementary Materials for full heat map). This analysis depicts how prioritized environmental attributes are distributed among stakeholder groups and represents a novel approach in using the FEGS Scoping Tool data to illustrate shared interests among stakeholder groups.

Flooding is a top concern for most stakeholder groups, but especially for those that are in closer proximity, such as the County Agencies, compared to State and Federal Agencies. General Public has a wide variety of beneficiary roles (Figure 3) that result in being represented by a larger interest in edible fauna over flooding. Commuters are a stakeholder that has a singular beneficiary, Transporters of People, whose main concern for the decision was flooding (Table 3), which is evident in the very high value for flooding for Commuters. Industrial Timber Neighbors have beneficiary roles that were equally distributed among Foresters, Commercial/Industrial and Transporters of Goods, all of which shared flooding as the main attribute of concern.

## 4 Discussion

Identifying some stakeholders was straightforward, such as the groups that spearheaded the acquisition for the land at the restoration site and who are supporting the restoration and management. Setting the decision context was important, and necessary to keep focus on who and what interests and impacts to include. Since there were different adjacent land uses that have been or pose to be impacted by flood events and by management interventions, we included those distinct groups. Utilities were one group that was considered because the TEP managers wanted to include local utility providers that likely have infrastructure running through the site, though at the time they were not sure how the stakeholder may be impacted or what beneficiaries they would represent in the restoration project. This is an example of including a potential stakeholder that may not ultimately hold beneficiary roles in the decision context and is an approach relevant to other decision contexts. Furthermore, the tool preferences can be revised and re-calculated if the TEP restoration managers determine that Utilities have beneficiary roles or other interests in this case that should be accounted for in the environmental decision making. Using the FEGS Scoping Tool in an iterative nature is an approach relevant here and other decision contexts or applications.

There are environmental attributes and ecological processes that may be affected by restoration interventions, that impact less adjacent stakeholders, such as aquaculture operators and fishing industries (Commercial Community) who are downstream of the restoration site. Ultimately, we also decided to include a somewhat broad scope of stakeholders, including other actors who may potentially be able to exercise some influence or be impacted, such as the greater dairy community of Tillamook, three levels of government agencies (county, state, and federal), the public, and people who use the road that currently intersects the site, even if they do not reside in adjacent properties.

When examining each stakeholder group to characterize beneficiary roles, maintaining focus on the decision context was imperative because there are stakeholder groups with clear interests and benefits that do not pertain to the decision context at the restoration site. For example, there are commuters who may be interested in recreational benefits, making art inspired at the site, or care about the existence of a restored wetland, but we considered only what beneficiary roles are pertinent to a commuter. When identifying the beneficiary roles of a stakeholder, we considered how that stakeholder would interact with the TRW Restoration site, and not at other sites or as other roles that do not pertain to the defined stakeholder.

In characterizing what environmental attributes were of interest for each beneficiary role, we considered what aspects of the environment were important for how the beneficiary would be directly using, consuming, or appreciating nature at the restoration site. One area that was challenging to conceptually overcome during discussions was how coarse an environmental attribute could seem to the TEP restoration managers when considering how one element could represent different uses, aspects, or even interpretations of nature to different beneficiary roles. For example, edible fauna may be an attribute that signifies fish and shellfish of interest to an aquaculturist or fisherperson, but it may refer to the terrestrial community of waterfowl and mammals of interest to hunters. These nuances are difficult to parse out directly in the FEGS Scoping Tool, but they are important to keep track of in case they need to be parsed out in decision making, or perhaps in later stages of planning the restoration project, such as in setting goals, or identifying monitoring metrics and communication strategies. Thus, the TEP restoration managers were also interested in seeing how environmental attribute interests are distributed among stakeholder groups and the narrower classification of beneficiary groups. Being able to visualize those more individualized results can help to transparently interpret the nuances of environmental attributes for both stakeholder and beneficiary groups. This visualization of results can be useful for other decision contexts or applications of the tool.

There were 21 total beneficiary roles and 43 environmental attributes identified, so interpreting and taking into account how each track to a stakeholder’s interest may seem challenging to incorporate into discussions and decision making. To focus on the beneficiaries and environmental attributes most prominently represented and shared by stakeholders, we set a threshold value of 3.00 or greater for a more detailed analysis (see Supplementary Materials for full results of Tables 3, 4). A value of 3.00 or more was chosen because that includes a wide range of result values, up to 30.75 for Flooding. Other attributes below a result of 3.00 may yet be important to consider (for more details, the reader is directed to Hernandez et al., 2022). Despite using an abbreviated set of environmental attributes distributed across stakeholder and beneficiary groups, the full suite could be used to communicate the results of this analysis or be used to widen the scope of considerations in the decision-making process.

This study demonstrated the transferability of the FEGS Scoping Tool and NESCS Plus as decision-informing tools to other applications. Many of the decisions made about how to utilize the tool are relevant to other decision contexts or applications of the tool. Furthermore, as this tool can be applied iteratively, as new information about interested stakeholder groups and their preferences can be updated into the tool when new knowledge is obtained. The tool itself can be used as part of a participatory exercise to more directly discover what attributes are of most common interest among stakeholders, and those attributes could be suggested to closely incorporate into decision making and throughout the process of restoration and monitoring. Since flood damage is a current reality and flood events are projected to increase damage to the road that bisects the TRW Restoration site, it was no surprise that flooding was the top attribute of concern. As such, we suggest that if TEP restoration managers, or involved stakeholders, are interested in examining how the cadre of other benefits and environmental attribute interests overlap without the obvious flooding concern, an alternative application of the FEGS Scoping Tool could be run without considering flooding to allow the results to highlight the degree of shared interests among other attributes. This alternative, iterative application of the tool could also be a way to examine how interests would differ in a situation where flooding was not a collective concern.

The FEGS Scoping Tool provided a methodical way to critically think of what boundaries to set and bend when conceptualizing what beneficiary roles a stakeholder group was interested in with regard to the restoration site and decision context, and what attributes are needed to realize the benefits of interest. This research used a novel application to explore stakeholder interests and dynamics, tying ecosystem services and the associated human well-being endpoints into a restoration context. This study also used the data inputs of the FEGS Scoping Tool to conduct an additional analysis to convey the connections between stakeholders, beneficiaries, and environmental attributes using a heat map communication approach. The use of a heat map to visually communicate results can be valuable in other decision contexts or applications of the tool. The types of results can be used to communicate risk, potential impacts, or can target communications about progress in restoration and monitoring to the specific concerns of stakeholders and their interests in the ways they directly use, enjoy, or consume aspects of the environment.

## 5 Conclusion

The FEGS Scoping Tool application for the TRW Restoration project was conducted through a series of virtual conversations between TEP restoration managers and EPA scientists before the tool became publicly available. Early stages of the restoration project planning make an ideal time to use the FEGS Scoping Tool to explore the social-ecological interests of people who may be affected by the project. The tool created an opportunity to use a multi-criteria decision approach to transparently identify priorities rather than allow a subset of stakeholders to dominate decisions because they are the most vocal. This approach is especially important when the stakeholder dynamics and interests require transparency and an equitable approach for community support. The analysis from the tool also elucidated overlapping interests among groups that may not have been realized by the TEP restoration managers elsewise.

In this study, the FEGS Scoping Tool was used to examine what environmental attributes to prioritize in the TRW Restoration site considering restoration scenarios that either upgrade or replace the currently existing road. A limitation of this approach is that results reflect the inputs according to the knowledge of TEP restoration managers at that point in time, thus a risk of result bias toward the assumptions of stakeholders’ interests from the viewpoint of TEP restoration managers. The results are intended to inform restoration planning discussions with stakeholders, so inaccuracies may become revealed in the course of those discussions. The data inputs for this application of the FEGS Scoping Tool can then be revised to reflect the updated understanding of stakeholders’ common ecological interests. Further, the tool could be used in a participatory, iterative fashion with direct input from stakeholders to allow them to make sure their groups and perspectives are most accurately represented.

The results of the FEGS Scoping Tool and heat map analyses can be used to identify what benefits and concerns are of greatest interest to stakeholders and is transferable to other decision contexts and scenarios. This can inform the process of identifying social-ecological goals for the project. The novel application of heat maps in this study can be especially useful in identifying the nuances of environmental attributes of interest to specific stakeholders, which may be useful in developing communication strategies with non-expert audiences. Overall, this can help build trust with the public and community leaders.

## Supplementary Material

Supplement1

## Figures and Tables

**TABLE 1:** Stakeholder groups for the Tillamook River Wetlands Restoration project and a brief description of who they represent.

Stakeholder	Description of the Group
**NCLC Site Landowners**	The North Coast Land Conservancy is a non-profit conservation organization. Primary landowners of the site. They require wetland restoration as part of the property acquisition.
**TEP & Partners**	The Tillamook Estuaries Partnership, and other organizations involved in facilitating the decision making, restoration implementation, and management/monitoring. (TEP helps steer project, with less say than landowners.)
**Funders**	Organizations that fund site acquisition and restoration interventions. Includes Oregon Watershed Enhancement Board, private donors.
**Rural Resident Neighbors**	Residents who live on adjacent properties and have direct access to Burton Fraser Road. Excludes the Tillamook Shooters Association.
**Tillamook Shooters Association**	Landowner that sold the property; owners of adjacent property with interest to create a hunting/gun club and who share wetland habitat with the site.
**Industrial Timber Neighbors**	Non-residential, for-profit timber growth forests in adjacent property lands. Potential decision outcomes may require additional small land acquisitions from them.
**Industrial Dairy Neighbors**	Adjacent dairy operators who may be directly impacted from decision outcome.
**Commercial Community**	Other commerce – fishing industry, aquaculture operators that stand to be impacted from downstream effects due to decision outcome. Rock quarry in the greater neighborhood uses Burton-Fraser Road occasionally; they are not expected to receive other direct ecological benefits from the site.
**Dairy Community**	Represents the influence and interests of the broader coalition of dairy operators/farmers and the dairy industry in Tillamook Bay.
**Utilities**	Added to consider roles of local cable and electricity providers who may have infrastructure in/near the site, although no services infrastructure was known to exist in the immediate restoration site at the time of discussion.
**Commuters**	Locals who use the roads in question on a frequent basis to commute to and from adjacent communities.
**General Public**	Any resident within the county who can comment on the decision process or comment on the transportation related decisions and [collectively] influence.
**County Agencies**	Public Works, planning commissions. Will be very involved in road maintenance and permitting and planning potential road infrastructure changes.
**State Agencies**	Includes Oregon Department of Fish and Wildlife. Have permitting roles with interests in recreation (angling, hunting, etc.) and conservation. There is interest in seeing research done at this site.
**Federal Agencies**	Includes the National Oceanic and Atmospheric Administration, the U.S. Fish and Wildlife Service, the U.S. Army Corps of Engineers. Overall, have permitting and management roles, comment on Clean Water Act regulations, with missions to sustainably manage natural resources for existence, current and future benefit/use.

**TABLE 2:** Weights assigned to each criterion to determine the decision context.

Criterion	Weight
Level of **Influence**	100
**Rights**	100
Magnitude and Probability of **Impact**	90
**Proximity**	90
**Economic Interest**	70
**Urgency** and Temporal Immediacy	65
Level of **Interest**	50
**Fairness**	50
**Underrepresented & Underserved Populations**	10

Weights can range 0–100; criteria with a weight of 100 are the most important, and other criteria are subsequently weighted relative to those. Bolded word(s) for each criterion correspond to how each is summarized in Figure 4.

**TABLE 3:** Results of beneficiary interests for each of the top nine environmental attributes.

Beneficiary Classes and Subclasses																						
	Agricultural				Commercial/Industrial		Municipal/Residential		Transportation		subsistence	Recreational					Inspirational		Learning		Non-use	
Environmental attributes	Livestock grazers	Aquaculturalists	Farmers	Foresters	Timber/Fiber/Ornamental extractors	Commercial property owners	Residential property owners	Public Sector Property Owners	Transporters of goods	Transporters of people	Water subsisters	Experiencers/viewers	Food pickers/gatherers	Hunters	Anglers	Boaters	Spiritual and ceremonial participants	Artists	Students and educators	Researchers	People who care	TOTAL
Flooding	0.34	0.15	0.31	1.88	1.88	0.65	0.92	2.93	8.38	10.03	0.00	0.05	0.00	0.00	0.00	0.24	0.00	0.00	0.60	0.58	1.81	30.75
Edible Fauna	0.00	0.00	0.00	0.00	0.00	0.00	0.00	0.00	0.00	0.00	0.00	0.00	0.00	3.91	2.85	0.00	0.00	0.00	0.00	0.00	1.13	7.89
Ecological Condition	0.19	0.17	0.17	0.00	0.00	0.36	0.00	0.00	0.00	0.00	0.00	0.00	0.00	0.00	0.00	0.00	0.05	0.00	4.03	0.58	1.81	7.35
Water Quality	0.34	0.15	0.31	0.00	0.00	0.65	0.00	0.00	0.00	0.00	1.95	0.00	0.00	0.00	0.00	0.00	0.00	0.00	0.43	0.58	1.13	5.53
Viewscapes	0.00	0.00	0.00	0.00	0.00	0.00	0.89	0.00	0.00	0.53	0.00	1.08	0.00	0.00	0.00	0.00	0.05	0.09	0.26	0.00	1.81	4.70
Open Space	0.00	0.00	0.00	0.00	0.00	0.00	0.89	0.00	0.00	0.00	0.00	0.54	0.00	0.43	0.00	0.24	0.05	0.05	0.26	0.00	1.81	4.27
Fauna Community	0.00	0.15	0.00	0.00	0.00	0.00	0.21	0.00	0.00	0.00	0.00	0.81	0.00	0.00	0.00	0.00	0.00	0.09	0.86	0.83	1.13	4.08
Water Quantity	0.32	0.15	0.29	0.00	0.00	0.61	0.00	0.00	0.00	0.00	1.30	0.05	0.00	0.00	0.00	0.24	0.00	0.00	0.00	0.58	0.00	3.55
Flora Community	0.00	0.15	0.00	0.00	0.00	0.00	0.00	0.00	0.00	0.00	0.00	0.54	0.12	0.00	0.00	0.00	0.00	0.09	0.86	0.58	1.13	3.47

The last column is the total result score for each attribute. For visual ease, individual result values are highlighted, high-to-low, with the darkest to lightest shading as follows:

**Table:** 

Result	> 4.00	2.00 to 3.99	1.00 to 1.99	0.50 to 0.99	0.10 to 0.49	< 0.10

**TABLE 4:** Results of stakeholder interests for each of the top nine environmental attributes.

	Stakeholders															
Env. Attributes	County Agencies	Tillamook Shooters Association	Rural Resident Neighbors	NCLC Landowners	TEP & Partners	Industrial Timber Neighbors	Industrial Dairy Neighbors	Funders	Commuters	State Agencies	Federal Agencies	Dairy Community	Utilities	Commercial Community	General Public	Total
Flooding	4.35	2.34	0.97	0.47	0.47	5.58	2.44	0.45	6.97	0.39	0.39	1.10	0.00	4.34	0.51	30.75
Edible Fauna	0.42	0.87	0.25	0.47	0.47	0.00	0.00	0.83	0.00	1.40	1.39	0.31	0.00	0.00	1.48	7.89
Ecological Condition	0.12	1.20	0.03	1.25	1.25	0.18	0.52	0.99	0.00	0.39	0.39	0.50	0.00	0.17	0.37	7.35
Water Quality	0.08	0.17	1.78	0.34	0.34	0.32	0.94	0.28	0.00	0.27	0.28	0.31	0.00	0.15	0.26	5.53
Viewscapes	0.17	0.46	1.09	0.30	0.30	0.00	0.00	0.31	0.37	0.45	0.42	0.50	0.00	0.00	0.33	4.70
Open Space	0.13	0.34	1.01	0.31	0.31	0.00	0.00	0.37	0.00	0.45	0.42	0.50	0.00	0.00	0.42	4.27
Fauna Community	0.13	0.44	0.36	0.55	0.55	0.00	0.00	0.47	0.00	0.42	0.44	0.31	0.00	0.15	0.27	4.08
Water Quantity	0.03	0.01	1.20	0.16	0.16	0.30	0.89	0.17	0.00	0.09	0.11	0.00	0.00	0.15	0.27	3.55
Flora Community	0.10	0.39	0.11	0.49	0.49	0.00	0.00	0.44	0.00	0.34	0.35	0.31	0.00	0.15	0.28	3.47

The last column is the total result score for each attribute. For visual ease, individual result values are highlighted, high-to-low, with the darkest to lightest shading as follows:

**Table:** 

Result	>2.00	1.0 to 1.999	0.5 to 0.999	0.1 to 0.499	<0.099

## Data Availability

The original contributions presented in the study are included in the article/Supplementary Material. Further inquiries can be directed to the corresponding author.

## Abbreviations:

EPA: U.S. Environmental Protection Agency
ES: Ecosystem services
FEGS: Final Ecosystem Goods and Services
MCDA: Multi-criteria decision analysis
OWEB: Oregon Watershed Enhancement Board
TEP: Tillamook Estuary Partnership
TRW: Tillamook River Wetlands
