# Supplementary material for: Connecting stakeholder priorities and desired environmental attributes for wetland restoration using ecosystem services and a heat map analysis for communications
Source: Front Ecol Evol. Author manuscript; Available in PMC 2025 Mar 27. (PMC11018255; doi:10.3389/fevo.2024.1290090)
Supplement: Supplement1 [file NIHMS1981740-supplement-Supplement1.zip › DataSheet_1_Connecting stakeholder priorities and desired environmental attributes for wetland restoration using ecosystem services and a heat map ana.pdf]

United States Environmental Protection Agency

EJScreen Report (Version 2.11)

2 miles Ring around the Area, OREGON, EPA Region 10

Approximate Population: 4,395

Input Area (sq. miles): 16.12

TRW Restoration Site

Selected Variables	State Percentile	USA Percentile
**Environmental Justice Indexes**		
Particulate Matter 2.5 EJ index	3	10
Ozone EJ index	5	2
Diesel Particulate Matter EJ index*	22	9
Air Toxics Cancer Risk EJ index*	21	35
Air Toxics Respiratory HI EJ index*	19	45
Traffic Proximity EJ index	71	62
Lead Paint EJ index	88	74
Superfund Proximity EJ index	34	31
RMP Facility Proximity EJ index	70	57
Hazardous Waste Proximity EJ index	14	4
Underground Storage Tanks EJ index	77	69
Wastewater Discharge EJ index	25	15

EJ Indexes - The EJ indexes help users screen for potential EJ concerns. To do this, the EJ index combines data on low income and people of color populations with a single environmental indicator.

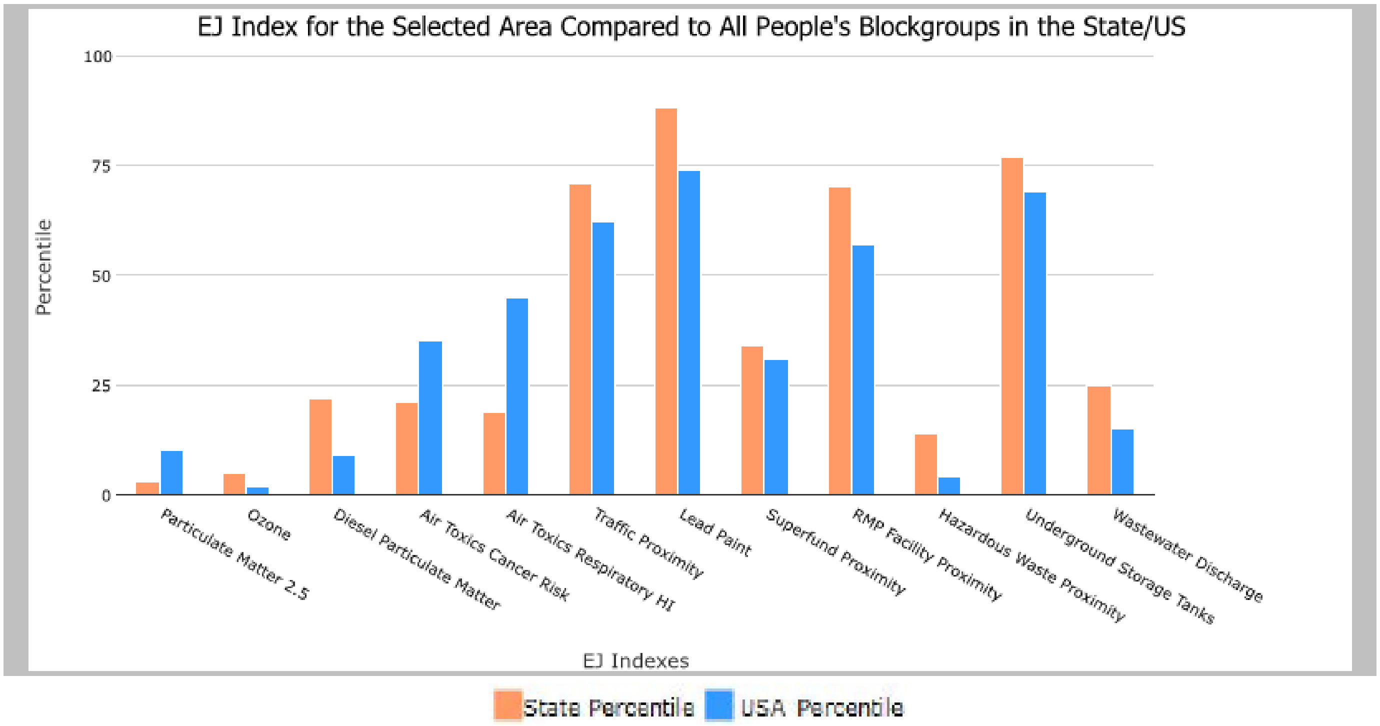

*Diesel particular matter, air toxics cancer risk, and air toxics respiratory hazard index are from the EPA's Air Toxics Data Update, which is the Agency's ongoing, comprehensive evaluation of air toxics in the United States. This effort aims to prioritize air toxics, emission sources, and locations of interest for further study. It is important to remember that the air toxics data presented here provide broad estimates of health risks over geographic areas of the country, not definitive risks to specific individuals or locations. Cancer risks and hazard indices from the Air Toxics Data Update are reported to one significant figure and any additional significant figures here are due to rounding. More information on the Air Toxics Data Update can be found at: https://www.epa.gov/haps/air-toxics-data-update.

EJScreen Report (Version 2.11)

2 miles Ring around the Area, OREGON, EPA Region 10

Approximate Population: 4,395

Input Area (sq. miles): 16.12

TRW Restoration Site

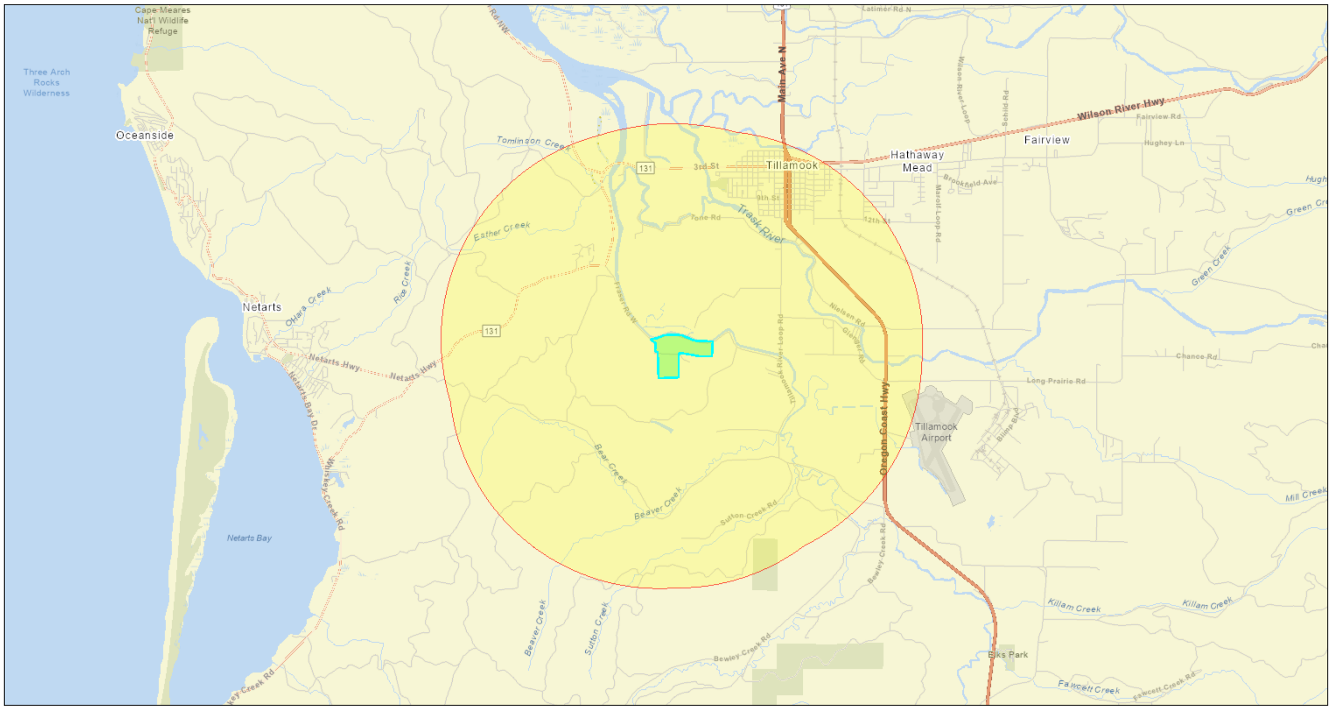

May 26, 2023

TRW Restoration Site

1:72,224

0 0.5 1 2 mi
0 1 2 4 km

Oregon State Parks, State of Oregon GEO, Esri, HERE, Garmin, SafeGraph, GeoTechnologies, Inc, METI/NASA, USGS, Bureau of Land Management, EPA, NPS, USDA

Sites reporting to EPA	
Superfund NPL	0
Hazardous Waste Treatment, Storage, and Disposal Facilities (TSDF)	0

EJScreen Report (Version 2.11)

2 miles Ring around the Area, OREGON, EPA Region 10

Approximate Population: 4,395

Input Area (sq. miles): 16.12

TRW Restoration Site

Selected Variables	Value	State Avg.	%ile in State	USA Avg.	%ile in USA
**Pollution and Sources**					
Particulate Matter 2.5 (µg/m^3)	6.02	8.69	1	8.67	4
Ozone (ppb)	28.8	37	2	42.5	1
Diesel Particulate Matter* (µg/m^3)	0.0521	0.337	12	0.294	<50th
Air Toxics Cancer Risk* (lifetime risk per million)	20	32	19	28	<50th
Air Toxics Respiratory HI*	0.28	0.47	16	0.36	<50th
Traffic Proximity (daily traffic count/distance to road)	400	660	63	760	61
Lead Paint (% Pre-1960 Housing)	0.5	0.24	81	0.27	73
Superfund Proximity (site count/km distance)	0.02	0.081	19	0.13	17
RMP Facility Proximity (facility count/km distance)	0.26	0.78	51	0.77	45
Hazardous Waste Proximity (facility count/km distance)	0.02	1.6	7	2.2	2
Underground Storage Tanks (count/km^2)	4.1	3.8	68	3.9	73
Wastewater Discharge (toxicity-weighted concentration/m distance)	6.4E-06	0.0046	25	12	16
**Socioeconomic Indicators**					
Demographic Index	35%	27%	75	35%	58
Supplemental Demographic Index	17%	13%	78	15%	69
People of Color	24%	25%	60	40%	44
Low Income	45%	29%	81	30%	75
Unemployment Rate	6%	5%	62	5%	64
Limited English Speaking Households	2%	2%	74	5%	65
Less Than High School Education	11%	9%	69	12%	59
Under Age 5	4%	5%	45	6%	41
Over Age 64	17%	18%	51	16%	58
Low Life Expectancy	21%	19%	77	20%	66

EJScreen is a screening tool for pre-decisional use only. It can help identify areas that may warrant additional consideration, analysis, or outreach. It does not provide a basis for decision-making, but it may help identify potential areas of EJ concern. Users should keep in mind that screening tools are subject to substantial uncertainty in their demographic and environmental data, particularly when looking at small geographic areas. Important caveats and uncertainties apply to this screening-level information, so it is essential to understand the limitations on appropriate interpretations and applications of these indicators. Please see EJScreen documentation for discussion of these issues before using reports. This screening tool does not provide data on every environmental impact and demographic factor that may be relevant to a particular location. EJScreen outputs should be supplemented with additional information and local knowledge before taking any action to address potential EJ concerns.

EJScreen Report (Version 2.11)

2 miles Ring around the Area, OREGON, EPA Region 10

Approximate Population: 4,395

Input Area (sq. miles): 16.12

TRW Restoration Site

Selected Variables	State Percentile	USA Percentile
**Supplemental Indexes**		
Particulate Matter 2.5 Supplemental Index	2	8
Ozone Supplemental Index	4	0
Diesel Particulate Matter Supplemental Index*	19	7
Air Toxics Cancer Risk Supplemental Index*	17	36
Air Toxics Respiratory HI Supplemental Index*	15	48
Traffic Proximity Supplemental Index	72	69
Lead Paint Supplemental Index	88	80
Superfund Proximity Supplemental Index	29	28
RMP Facility Proximity Supplemental Index	72	61
Hazardous Waste Proximity Supplemental Index	8	2
Underground Storage Tanks Supplemental Index	78	75
Wastewater Discharge Supplemental Index	22	14

Supplemental Indexes - The supplemental indexes offer a different perspective on community-level vulnerability. They combine data on low-income, limited English speaking, less than high school education, unemployed, and low life expectancy populations with a single environmental indicator.

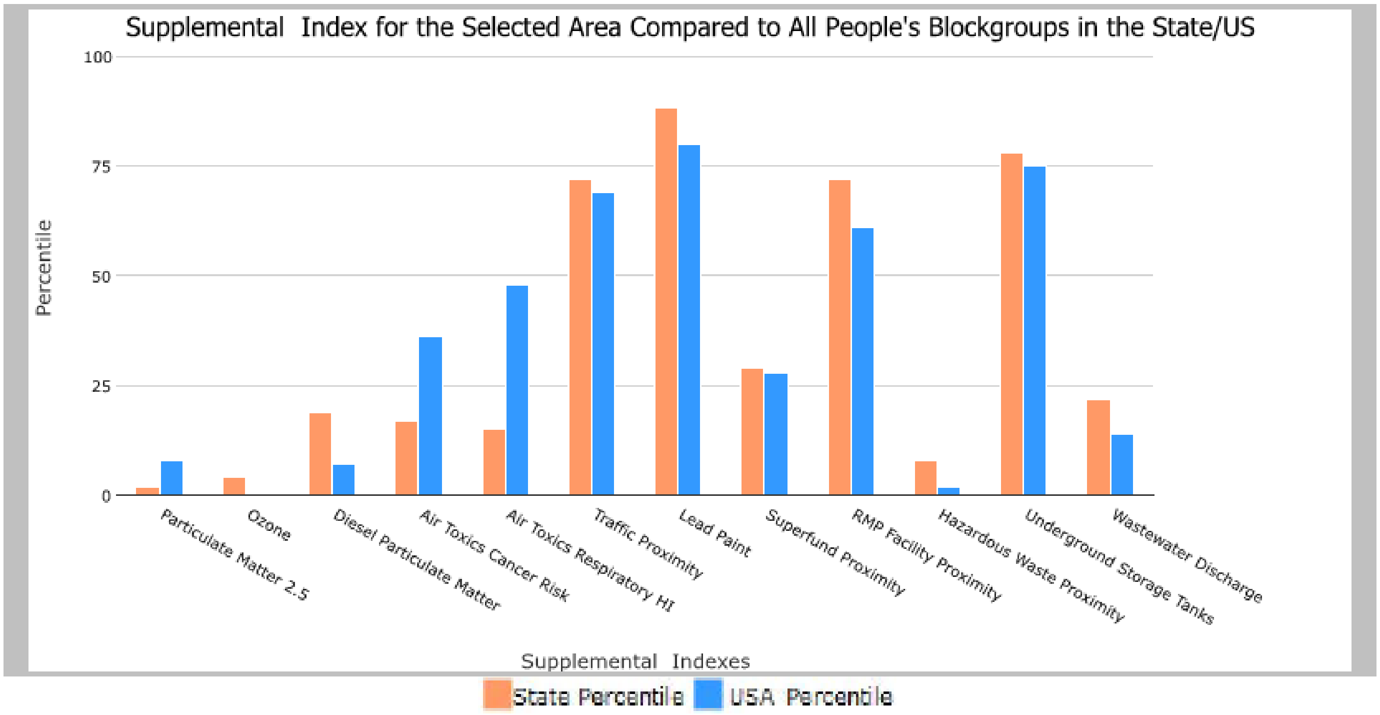

This report shows the values for environmental and demographic indicators, EJScreen indexes, and supplemental indexes. It shows environmental and demographic raw data (e.g., the estimated concentration of ozone in the air), and also shows what percentile each raw data value represents. These percentiles provide perspective on how the selected block group or buffer area compares to the entire state, EPA region, or nation. For example, if a given location is at the 95th percentile nationwide, this means that only 5 percent of the US population has a higher block group value than the average person in the location being analyzed. The years for which the data are available, and the methods used, vary across these indicators. Important caveats and uncertainties apply to this screening-level information, so it is essential to understand the limitations on appropriate interpretations and applications of these indicators. Please see EJScreen documentation for discussion of these issues before using reports. For additional information, see: www.epa.gov/environmentaljustice.
