# Supplementary material for: Connecting stakeholder priorities and desired environmental attributes for wetland restoration using ecosystem services and a heat map analysis for communications
Source: Front Ecol Evol. Author manuscript; Available in PMC 2025 Mar 27. (PMC11018255; doi:10.3389/fevo.2024.1290090)
Supplement: Supplement1 [file NIHMS1981740-supplement-Supplement1.zip › DataSheet_2_Connecting stakeholder priorities and desired environmental attributes for wetland restoration using ecosystem services and a heat map ana.docx]

1. **Detailed math used in calculating the outputs of the FEGS Scoping Tool and the heat map crosswalk (Excerpt from the FEGS Scoping Tool Manual, Sharpe 2021)**

The FEGS Scoping Tool is a multi-criteria decision analysis tool that uses the method of ranking the alternatives on the sum of weighted criteria.

Multi-criteria decision analysis (MCDA) is a formal approach to problem solving that attempts to represent the decision goals in terms of explicitly evaluated criteria (Stewart 1992). The MCDA approach is a transparent method of informing decision making; it formalizes key criteria, explicitly states priorities, and permits easy replication and justification of results. Multi-criteria decision analysis leaves a ‘paper trail’ of the criteria & relative weights used when evaluating alternatives.

The MCDA approach allows users to prioritize investments of limited resources and evaluate their choices relative to strategic plans or other higher-level priorities. It allows for a formal exploration of trade-offs (e.g., using different sets of weights to compare the priorities of different groups, manipulating the weights to explore the sensitivity of the tool output, etc.).

Finally, MCDA tools can be used in an iterative and participatory fashion, involving representatives and feedback from various stakeholder groups, providing a clear pathway for stakeholder engagement in the process.

There are seven steps in a generic MCDA process. Different methods will approach these steps in a variety of ways, but the essentials will remain the same. This tool addressed these steps in the following manner:

1. *Identify decision objectives* - The overall objective of the tool is to prioritize the relevant stakeholder groups for a decision with an environmental context and, building on that initial prioritization, also prioritize the beneficiary groups and key environmental attributes for that decision context.
2. *Identify decision criteria* - Decision criteria are the factors that should be considered when evaluating alternatives to ensure project objectives are met. These are the stakeholder prioritization criteria laid out in the tool.
3. *Identify metrics and value functions for the criteria* - Metrics are measurable properties that are used to quantify and compare the performance of the alternatives for each criterion. These criteria should be as objective as possible. Value functions then relate performance scores on each metric to value on the criteria by converting the alternative’s scores on each metric into a numerical value that captures the relative performance on a fixed scale. These are contained in the Stakeholder scoring step of the tool.
4. *Develop a range of alternatives* - These are the alternatives that will be scored by the metrics for each criterion and ultimately prioritized by the tool. In this case, the alternatives are the stakeholder groups that are identified and input into the tool by the users.
5. *Weight the relative importance of the criteria* - Weights capture the preference for, or importance of, each decision criterion relative to the others. This step is entirely subjective and aimed at clearly expressing the decision maker’s priorities. User input is the sole source of weights in this tool.
6. *Evaluate the performance of each alternative with respect to each of the metrics and assign value scores* - In this step, the decision maker simply uses the metrics identified in Step 3 and the value functions developed in Step 4, to assign scores to all criteria for each of the alternatives identified in Step 5. This is what the user is doing in the Stakeholder step of the tool.
7. *Calculate the MCDA value* - In this step, the weights and the value scores are used to calculate the overall MCDA value of each alternative. The tool performs these calculations displaying the MCDA value as the bar chart output of the Stakeholder step.

The FEGS Scoping Tool, however, does not merely prioritize the stakeholder groups. It uses that initial prioritization to subsequently also prioritize beneficiary groups, and then environmental attributes. It does this by taking an approach we are calling a tiered MCDA. This approach uses the output from the stakeholder prioritization MCDA as input in a beneficiary prioritization MCDA, and then the output from the beneficiary prioritization as input in an environmental attribute prioritization. This is described in Table A-1.

Table A-1. The MCDA steps in each of the three tiers of the FEGS Scoping Tool. The colored boxes indicate how the output of one tier is used as an input in the next. The output from Tier 1 (red box) is used as an input in Tier 2 (red box). The output from Tier 2 (orange box) is used as an input in Tier 3 (orange box).

| MCDA Steps | Tier 1: Stakeholders | Tier 2: Beneficiaries | Tier 3: Attributes |
| --- | --- | --- | --- |
| 1. Objective | Prioritize stakeholders | Prioritize beneficiaries | Prioritize environmental attributes |
| 1. Decision criteria | Used when prioritizing stakeholder groups – supplied by the tool | Used when prioritizing beneficiaries – the stakeholder groups themselves are used as these criteria (i.e., which beneficiary groups are relevant to stakeholders) | Used when prioritizing attributes – the beneficiary groups are used as these criteria (i.e., which attributes are relevant to beneficiaries) |
| 1. Metrics & Value functions | Used to score each stakeholder group for each criterion – supplied by the tool | Used to score each beneficiary group for each stakeholder group – this is done in the Beneficiary step of the tool when users are asked to identify those beneficiary categories found within each stakeholder group | Used to score each attribute for each beneficiary group – this is done in the Attribute step of the tool when users are asked to identify attributes of concern for each beneficiary group |
| 1. Alternatives | Stakeholders identified by tool users | Beneficiary list from the FEGS Classification System | Attribute list from the FEGS Classification System |
| 1. Weighting | Done by users in the first step of the tool | Stakeholder MCDA values from the output of Tier 1 are used as weights in this step | Beneficiary MCDA values from the output of Tier 2 are used as weights in this step |
| 1. Score alternatives | User input at the Stakeholder step | User input at the Beneficiary step | User input at the Attribute step |
| 1. Calculate value | Output at Stakeholder step | Output at Beneficiary step | Output at Attribute step |

The FEGS Scoping Tool calculates a value for each alternative (e.g., a specific stakeholder, beneficiary, or attribute) by summing the weighted scores for each alternative. The value, *y(i)*, of an alternative, *i*, is calculated as:

$$y\left( i \right)= \sum_{m=1}^{M} w_{m}z_{i}$$

where *M* is the number of possible metrics for which *i* can be scored, *w_m_* is the weight given to each criterion, and *z_i_* is the score of alternative *i* on metric *m*. The value, *y(i)*, is then normalized, *n(i)*, to a 0 – 100 scale by dividing *y(i)* by the sum of all weights:

$$n\left( i \right)= \frac{y(i)}{\sum_{m=1}^{M} w_{m}}$$

The same calculations are used for each tier of the FEGS Scoping Tool. The output values, *n(i)*, from Tier 1 are then used as the weights, *w_m_*, for Tier 2. Subsequently, the output values, *n(i)*, from Tier 2 are used as the weights, *w_m_*, for Tier 3.

Another way to look at the FEGS Scoping Tool calculations is as follows:

In step 1, the FEGS Scoping Tool calculates a value for each stakeholder by summing the weighted scores for each stakeholder. The value, *v(s)*, of a stakeholder, *s*, is calculated as:

$$v\left( s \right)= \sum_{c=1}^{C} w_{c}z_{s}$$

where *C* is the number of possible stakeholder prioritization criteria for which *s* can be scored, *w_c_* is the weight given to each criterion, and *z_s_* is the score of stakeholder group, *s,* on criterion *c*. The value, *v(s)*, is then normalized, *n(s)*, to a 0 – 100 scale by dividing *n(s)* by the sum of all weights:

$$n\left( s \right)= \frac{v(s)}{\sum_{c=1}^{C} w_{c}}$$

In step 2, the FEGS Scoping Tool builds upon step 1 and uses the results of step 1 as the weights in step 2. In this step, the tool calculates a value for each beneficiary by summing the weighted scores for each beneficiary. The value, *v(b)*, of a beneficiary, *b*, is calculated as:

$$v\left( b \right)= \sum_{s=1}^{S} w_{n(s)}z_{b}$$

where *S* is the number of possible stakeholder groups for which *b* can be scored (i.e., the number of stakeholder groups that might contain that beneficiary group in some way), *w_n(s)_* is the weight given to each stakeholder group (i.e., their normalized value, *n(s)*, from step 1), and *z_b_* is the score of beneficiary *b* for stakeholder group *s*.

The value, *v(b)*, is then normalized, *n(b)*, to a 0 – 100 scale by dividing *v(b)* by the sum of all weights:

$$n\left( b \right)= \frac{v(b)}{\sum_{s=1}^{S} w_{n(s)}}$$

In step 3, the FEGS Scoping Tool builds upon step 1 and uses the results of step 2 as the weights in step 3. In this step, the tool calculates a value for each beneficiary by summing the weighted scores for each beneficiary. The value, *v(a)*, of an attribute, *a*, is calculated as:

$$v\left( a \right)= \sum_{b=1}^{B} w_{n(b)}z_{a}$$

where *B* is the number of possible beneficiary groups for which *a* can be scored (i.e., the number of beneficiary groups that might need that attribute in some way), *w_n(b)_* is the weight given to each beneficiary group (i.e., their normalized value, *n(b)*, from step 2), and *z_a_* is the score of attribute *a* for beneficiary group *b*. The value, *v(a)*, is then normalized, *n(a)*, to a 0 – 100 scale by dividing *v(a)* by the sum of all weights:

$$n\left( a \right)= \frac{v(a)}{\sum_{b=1}^{B} w_{n(b)}}$$

All three steps of the tool produce graphical outputs showing how the overall values of each alternative (Step 1, *n(s)*; Step 2, *n(b)*; Step 3, *n(a)*) are made up of their constituent weighted scores. Given the number of beneficiary groups, the final step of the tool sums the individual group into higher level beneficiary categories and displays *n(a)* as being made up of those summed and weighted scores. This was done to make the graphical output less complicated and more legible.

The two additional outputs referenced in the “Beneficiaries x Environmental attributes” and “Stakeholders x Environmental attributes” sections were created using the above math and the same inputs as were used in the tool. Those outputs differ from the tool as follows:

Beneficiaries x Environmental attribute: The Beneficiary X Environmental attribute crosswalk displays *n(a)* as being made of all the weighted scores, instead of having the weighted scores summed at the beneficiary category level.

Stakeholders x Environmental attributes: The Stakeholder X Environmental attribute crosswalk uses the above calculations to display *n(a)* in relationship to the stakeholder groups rather than the beneficiary groups. That was done by recalculating *n(a)*, of an attribute, *a*, as follows.

We begin by normalizing each weighted score making up the value, *v(a)*, of an attribute, *a*, instead of normalizing the value, *v(a)*, after the weighted scores are summed. This value, *n(ab)*, for attribute, *a*, and beneficiary, *b*, is calculated as:

$$n\left( ab \right)= z_{a}\frac{n(b)}{\sum_{b=1}^{B} n(b)}$$

where *z_a_* is the score of attribute *a* on beneficiary group *b*, *n(b)* is the normalized value for that beneficiary group.

We next calculate what proportion of beneficiary group value, *p(bs)*, results from each stakeholder group:

$$p\left( bs \right)= \frac{z_{b}}{\sum_{s=1}^{S} z_{b}}$$

where *z_b_* is the score of beneficiary group *b* for stakeholder group *s*.

The next step gives us the normalized value, *n(as)*, for attribute, *a*, and beneficiary, *b*, calculated as:

$$n\left( as \right)= \sum_{b=1}^{B} (n\left( ab \right)*p(bs))$$

where the normalized value for a specific beneficiary/attribute combination, *n(ab)*, is multiplied by, *p(bs)*, the proportion of that beneficiary value resulting from a stakeholder group, *s*, and summed across all beneficiary groups, *B*. The final step for this analysis, which once again gives us the normalized value, *n(a)*, of an attribute, this time displayed in relation to stakeholder groups is:

$$n\left( a \right)= \sum_{s=1}^{S} (n(as))$$

References:

Stewart, T.J. (1992). A critical survey of the status of multiple criteria decision making theory and practice. Omega 20:569-586.

Sharpe, L.M. (2021) FEGS Scoping Tool User Manual. U.S. Environmental Protection Agency, Gulf Breeze, FL, EPA/600/X-21/104.

1. **All input tables**

Input Tables for the FEGS Scoping Tool

**Table 1.** Stakeholder group scores for the decision criteria, as assigned by TEP Restoration Managers. This produces the relative ranking of stakeholder groups (Figure 3 in report).

| **Stakeholder Group** | **Magnitude & Probability of Impact** | **Level of Influence** | **Level of Interest** | **Urgency & Temporal Immediacy** | **Proximity** | **Economic Interest** | **Rights** | **Fairness** | **Underrepresented & Underserved Representation** |
| --- | --- | --- | --- | --- | --- | --- | --- | --- | --- |
| NCLC Site Landowners | 20 | 100 | 100 | 60 | 50 | 100 | 100 | 100 | 50 |
| TBNEP & Partners | 20 | 100 | 100 | 60 | 75 | 100 | 60 | 100 | 50 |
| Funders | 20 | 75 | 100 | 60 | 10 | 100 | 60 | 100 | 50 |
| Rural Resident Neighbors | 80 | 75 | 100 | 20 | 100 | 100 | 60 | 100 | 100 |
| Tillamook Shooters Association | 80 | 100 | 100 | 20 | 100 | 100 | 60 | 100 | 0 |
| Industrial Timber Neighbors | 50 | 100 | 100 | 20 | 75 | 100 | 20 | 100 | 0 |
| Industrial Dairy Neighbors | 35 | 75 | 100 | 20 | 75 | 100 | 20 | 100 | 0 |
| Commercial Community | 5 | 50 | 50 | 20 | 25 | 100 | 20 | 100 | 0 |
| Dairy Community | 0 | 75 | 100 | 20 | 50 | 100 | 20 | 100 | 0 |
| Utilities | 20 | 50 | 50 | 20 | 50 | 0 | 60 | 100 | 0 |
| Commuters | 80 | 75 | 100 | 20 | 100 | 0 | 20 | 100 | 0 |
| General Public | 5 | 50 | 50 | 20 | 50 | 0 | 20 | 100 | 0 |
| County Agencies | 80 | 100 | 100 | 40 | 75 | 100 | 100 | 100 | 50 |
| State Agencies | 0 | 100 | 100 | 40 | 50 | 100 | 20 | 100 | 50 |
| Federal Agencies | 0 | 100 | 100 | 40 | 50 | 100 | 20 | 100 | 50 |

Beneficiary Profiles

The representation and relative distribution of beneficiaries for each stakeholder group. Each stakeholder group has their own table with the distribution of beneficiary roles they represent. This information results in the relative beneficiary prioritization (Figure 5 in report).

**Table 2.** Beneficiary profile for the NCLC Site Landowners stakeholder group.

| **Beneficiary Category** | **Subcategory** | **Score** |
| --- | --- | --- |
| Recreational | Experiencers/Viewers | 3 |
|  | Food Pickers/Gatherers | 3 |
|  | Hunters | 3 |
|  | Anglers | 3 |
|  | Boaters | 2 |
| Inspirational | Spiritual and Ceremonial Participants | 3 |
|  | Artists | 3 |
| Learning | Students and Educators | 25 |
|  | Researchers | 25 |
| Non-Use | People Who Care | 30 |

**Table 3:** Beneficiary profile for the TBNEP & Partners stakeholder group.

| **Beneficiary Category** | **Subcategory** | **Score** |
| --- | --- | --- |
| Recreational | Experiencers/Viewers | 3 |
|  | Food Pickers/Gatherers | 3 |
|  | Hunters | 3 |
|  | Anglers | 3 |
|  | Boaters | 2 |
| Inspirational | Spiritual and Ceremonial Participants | 3 |
|  | Artists | 3 |
| Learning | Students and Educators | 25 |
|  | Researchers | 25 |
| Non-Use | People Who Care | 30 |

**Table 4.** Beneficiary Profile for the Funders stakeholder group.

| **Beneficiary Category** | **Subcategory** | **Score** |
| --- | --- | --- |
| Recreational | Experiencers/Viewers | 6 |
|  | Food Pickers/Gatherers | 6 |
|  | Hunters | 6 |
|  | Anglers | 6 |
|  | Boaters | 6 |
| Inspirational | Spiritual and Ceremonial Participants | 1 |
|  | Artists | 1 |
| Learning | Students and Educators | 19 |
|  | Researchers | 19 |
| Non-Use | People Who Care | 30 |

**Table 5.** Beneficiary profile for the Rural Resident Neighbors stakeholder group.

| **Beneficiary Category** | **Subcategory** | **Score** |
| --- | --- | --- |
| Agricultural | Livestock Grazers | 2 |
| Gov/Muni/Res | Residential Property Owners | 40 |
| Subsistence | Water Subsisters | 35 |
| Recreational | Experiencers/Viewers | 13 |
|  | Food Pickers/Gatherers | 2 |
|  | Hunters | 2 |
|  | Anglers | 2 |
| Inspirational | Spiritual and Ceremonial Participants | 2 |
| Non-Use | People Who Care | 2 |

**Table 6**. Beneficiary profile for the Tillamook Shooters Association stakeholder group.

| **Beneficiary Category** | **Subcategory** | **Score** |
| --- | --- | --- |
| Transportation | Transporters of People | 30 |
| Recreational | Experiencers / Viewers | 14 |
|  | Hunters | 13 |
| Learning | Students and Educators | 30 |
| Non-Use | People Who Care | 13 |

**Table 7.** Beneficiary profile for the Industrial Timber Neighbors stakeholder group.

| **Beneficiary Category** | **Subcategory** | **Score** |
| --- | --- | --- |
| Agricultural | Foresters | 25 |
| Commercial/ Industrial | Timber / Fiber / Ornamental Extractors | 25 |
|  | Commercial Property Owners | 25 |
| Transportation | Transporters of Goods | 25 |

**Table 8.** Beneficiary profile for the Industrial Dairy Neighbors stakeholder group.

| **Beneficiary Category** | **Subcategory** | **Score** |
| --- | --- | --- |
| Agricultural | Livestock Grazers | 25 |
|  | Farmers | 25 |
| Commercial/ Industrial | Commercial Property Owners | 25 |
| Transportation | Transporters of Goods | 25 |

**Table 9.** Beneficiary profile for the Commercial Community stakeholder group.

| **Beneficiary Category** | **Subcategory** | **Score** |
| --- | --- | --- |
| Agricultural | Aquaculturists | 30 |
| Transportation | Transporters of Goods | 70 |

**Table 10.** Beneficiary profile for the Dairy Community stakeholder group.

| **Beneficiary Category** | **Subcategory** | **Score** |
| --- | --- | --- |
| Transportation | Transporters of Goods | 10 |
| Non-Use | People Who Care | 90 |

**Beneficiary profile for the Utilities stakeholder**

This stakeholder group does not directly benefit from the ecosystem.

**Table 11.** Beneficiary profile for the Commuters stakeholder group.

| **Beneficiary Category** | **Subcategory** | **Score** |
| --- | --- | --- |
| Transportation | Transporters of People | 100 |

**Table 12.** Beneficiary profile for the General Public stakeholder group.

| **Beneficiary Category** | **Subcategory** | **Score** |
| --- | --- | --- |
| Transportation | Transporters of People | 4 |
| Subsistence | Water Subsisters | 4 |
| Recreational | Experiencers / Viewers | 12 |
|  | Food Pickers / Gatherers | 12 |
|  | Hunters | 12 |
|  | Anglers | 12 |
|  | Boaters | 12 |
| Inspirational | Spiritual and Ceremonial Participants | 8 |
|  | Artists | 8 |
| Learning | Students and Educators | 8 |
| Non-Use | People Who Care | 8 |

**Table 13.** Beneficiary profile for the County Agencies stakeholder group.

| **Beneficiary Category** | **Subcategory** | **Score** |
| --- | --- | --- |
| Gov/Muni/Res | Public Property Owners | 50 |
| Transportation | Transporters of Goods | 10 |
|  | Transporters of People | 10 |
| Recreational | Experiencers/Viewers | 3 |
|  | Hunters | 3 |
|  | Anglers | 3 |
| Learning | Researchers | 5 |
| Non-Use | People Who Care | 16 |

**Table 14.** Beneficiary profile for the State Agencies stakeholder group.

| **Beneficiary Category** | **Subcategory** | **Score** |
| --- | --- | --- |
| Recreational | Experiencers/Viewers | 10 |
|  | Hunters | 10 |
|  | Anglers | 10 |
| Learning | Researchers | 15 |
| Non-Use | People Who Care | 55 |

**Table 15.** Beneficiary profile for the Federal Agencies stakeholder group.

| **Beneficiary Category** | **Subcategory** | **Score** |
| --- | --- | --- |
| Recreational | Experiencers/Viewers | 10 |
|  | Hunters | 10 |
|  | Anglers | 10 |
| Learning | Researchers | 20 |
| Non-Use | People Who Care | 50 |

Environmental Attribute Profiles

The representation and relative distribution of environmental attributes for each beneficiary group. Each beneficiary group has their own table with the distribution of environmental attributes they need or care about. This information results in the relative environmental attribute prioritization (Figure 7 in report).

**Recreational Beneficiaries**

**Table 16:** Environmental Attribute profile for the Experiencers/Viewers beneficiary group.

| **Attribute Category** | **Attribute Subcategory** | **Score** |
| --- | --- | --- |
| Atmosphere | Air Quality | 8 |
| Water | Water Quantity | 1 |
|  | Water Movement | 1 |
| Fauna | Fauna Community | 15 |
|  | Charismatic Fauna | 5 |
|  | Rare Fauna | 2 |
|  | Spiritually/Culturally Important Fauna | 1 |
| Flora | Flora Community | 10 |
|  | Charismatic Flora | 1 |
|  | Rare Flora | 1 |
|  | Spiritually/Culturally Important Flora | 1 |
| Fungi | Fungal Community | 1 |
|  | Rare Fungi | 1 |
|  | Spiritually/Culturally Important Fungi | 1 |
| Composite (and Extreme Events) | Sounds | 10 |
|  | Scents | 5 |
|  | Viewscapes | 20 |
|  | Phenomena | 5 |
|  | Open Space | 10 |
|  | Flooding | 1 |

**Table 17:** Environmental Attribute profile for the Food Pickers/Gatherers beneficiary group.

| **Attribute Category** | **Attribute Subcategory** | **Score** |
| --- | --- | --- |
| Flora | Flora Community | 8 |
|  | Edible Flora | 15 |
|  | Medicinal Flora | 15 |
| Fungi | Fungal Community | 8 |
|  | Edible Fungi | 15 |
|  | Medicinal Fungi | 15 |
| Other Natural Components | Fiber Material Quality | 8 |
|  | Fiber Material Quantity | 8 |
|  | Presence of ONC for Artistic Use or Consumption | 8 |

**Table 18:** Environmental Attribute profile for the Hunters beneficiary group.

| **Attribute Category** | **Attribute Subcategory** | **Score** |
| --- | --- | --- |
| Fauna | Edible Fauna | 90 |
| Composite (and Extreme Events) | Open Space | 10 |

**Table 19:** Environmental Attribute profile for the Anglers beneficiary group.

| **Attribute Category** | **Attribute Subcategory** | **Score** |
| --- | --- | --- |
| Water | Water Movement | 10 |
| Fauna | Edible Fauna | 90 |

**Table 20.** Environmental Attribute profile for the Boaters beneficiary group.

| **Attribute Category** | **Attribute Subcategory** | **Score** |
| --- | --- | --- |
| Atmosphere | Wind Strength/Speed | 20 |
| Water | Water Quantity | 20 |
|  | Water Movement | 20 |
| Composite (and Extreme Events) | Open Space | 20 |
|  | Flooding | 20 |

**Inspirational Beneficiaries**

**Table 21.** Environmental Attribute profile for the Spiritual and Ceremonial Participants beneficiary group.

| **Attribute Category** | **Attribute Subcategory** | **Score** |
| --- | --- | --- |
| Atmosphere | Air Quality | 3 |
|  | Sunlight | 3 |
| Water | Water Movement | 4 |
| Fauna | Medicinal Fauna | 1 |
|  | Charismatic Fauna | 10 |
|  | Spiritually/Culturally Important Fauna | 10 |
| Flora | Medicinal Flora | 4 |
|  | Charismatic Flora | 5 |
|  | Spiritually/Culturally Important Flora | 10 |
| Fungi | Medicinal Fungi | 5 |
|  | Spiritually/Culturally Important Fungi | 10 |
| Other Natural Components | Presence of Other Natural Components for Artistic Use or Consumption | 5 |
| Composite (and Extreme Events) | Sounds | 5 |
|  | Scents | 5 |
|  | Viewscapes | 5 |
|  | Phenomena | 5 |
|  | Ecological Condition | 5 |
|  | Open Space | 5 |

**Table 22.** Environmental Attribute profile for the Artists beneficiary group.

| **Attribute Category** | **Attribute Subcategory** | **Score** |
| --- | --- | --- |
| Atmosphere | Sunlight | 3 |
| Water | Water Movement | 3 |
| Fauna | Fauna Community | 10 |
|  | Charismatic Fauna | 10 |
|  | Spiritually/Culturally Important Fauna | 3 |
| Flora | Flora Community | 10 |
|  | Charismatic Flora | 3 |
|  | Spiritually/ Culturally Important Flora | 3 |
| Fungi | Fungal Community | 5 |
|  | Spiritually/Culturally Important Fungi | 3 |
| Other Natural Components | Fiber Material Quality | 5 |
|  | Fiber Material Quantity | 5 |
|  | Mineral/Chemical Quality | 1 |
|  | Mineral/Chemical Quantity | 1 |
|  | Presence of Other Natural Components for Artistic Use or Consumption | 5 |
| Composite (and Extreme Events) | Sounds | 3 |
|  | Scents | 1 |
|  | Viewscapes | 10 |
|  | Phenomena | 10 |
|  | Open Space | 6 |

**Learning Beneficiaries**

**Table 23.** Environmental Attribute profile for the Students and Educators beneficiary group.

| **Attribute Category** | **Attribute Subcategory** | **Score** |
| --- | --- | --- |
| Water | Water Quality | 5 |
| Fauna | Fauna Community | 10 |
| Flora | Flora Community | 10 |
| Fungi | Fungal Community | 3 |
| Composite (and Extreme Events) | Viewscapes | 3 |
|  | Phenomena | 3 |
|  | Ecological Condition | 47 |
|  | Open Space | 3 |
|  | Flooding | 7 |
|  | Wildfire | 3 |
|  | Extreme Weather Events | 3 |
|  | Earthquakes | 3 |

**Table 24.** Environmental Attribute profile for the Researchers beneficiary group.

| **Attribute Category** | **Attribute Subcategory** | **Score** |
| --- | --- | --- |
| Soil | Soil Quality | 7 |
|  | Substrate Quality | 7 |
| Water | Water Quality | 7 |
|  | Water Quantity | 7 |
|  | Water Movement | 7 |
| Fauna | Fauna Community | 10 |
|  | Keystone Fauna | 10 |
|  | Rare Fauna | 10 |
| Flora | Flora Community | 7 |
|  | Keystone Flora | 7 |
|  | Rare Flora | 7 |
| Composite (and Extreme Events) | Ecological Condition | 7 |
|  | Flooding | 7 |

**Agricultural Beneficiaries**

**Table 25.** Environmental Attribute profile for the Livestock Grazers beneficiary group.

| **Attribute Category** | **Attribute Subcategory** | **Score** |
| --- | --- | --- |
| Soil | Soil Quality | 10 |
|  | Substrate Quality | 10 |
| Water | Water Quality | 18 |
|  | Water Quantity | 17 |
|  | Water Movement | 17 |
| Composite (and Extreme Events) | Ecological Condition | 10 |
|  | Flooding | 18 |

**Table 26.** Environmental Attribute profile for the Aquaculturalists beneficiary group.

| **Attribute Category** | **Attribute Subcategory** | **Score** |
| --- | --- | --- |
| Soil | Substrate Quality | 11 |
|  | Substrate Quantity | 11 |
| Water | Water Quality | 11 |
|  | Water Quantity | 11 |
|  | Water Movement | 11 |
| Fauna | Fauna Community | 11 |
| Flora | Flora Community | 11 |
| Composite (and Extreme Events) | Ecological Condition | 12 |
|  | Flooding | 11 |

**Table 27.** Environmental Attribute profile for the Farmers beneficiary group.

| **Attribute Category** | **Attribute Subcategory** | **Score** |
| --- | --- | --- |
| Soil | Soil Quality | 10 |
|  | Substrate Quantity | 10 |
| Water | Water Quality | 18 |
|  | Water Quantity | 17 |
|  | Water Movement | 17 |
| Composite (and Extreme Events) | Ecological Condition | 10 |
|  | Flooding | 18 |

**Table 28.** Environmental Attribute profile for the Foresters beneficiary group.

| **Attribute Category** | **Attribute Subcategory** | **Score** |
| --- | --- | --- |
| Composite (and Extreme Events) | Flooding | 100 |

**Government/Municipal/Residential Beneficiaries**

**Table 29.** Environmental Attribute profile for the Residential Property Owners beneficiary group.

| **Attribute Category** | **Attribute Subcategory** | **Score** |
| --- | --- | --- |
| Fauna | Fauna Community | 6 |
|  | Charismatic Fauna | 6 |
| Composite (and Extreme Events) | Sounds | 6 |
|  | Scents | 6 |
|  | Viewscapes | 25 |
|  | Open Space | 25 |
|  | Flooding | 26 |

**Table 30.** Environmental Attribute profile for the Public Property Owners beneficiary group.

| **Attribute Category** | **Attribute Subcategory** | **Score** |
| --- | --- | --- |
| Composite (and Extreme Events) | Flooding | 60 |
|  | Earthquakes | 40 |

**Subsistence Beneficiaries**

**Table 31.** Environmental Attribute profile for the Water Subsisters beneficiary group.

| **Attribute Category** | **Attribute Subcategory** | **Score** |
| --- | --- | --- |
| Water | Water Quality | 60 |
|  | Water Quantity | 40 |

**Commercial/Industrial Beneficiaries**

**Table 32.** Environmental Attribute profile for the Timber/Fiber/Ornamental Extractors beneficiary group.

| **Attribute Category** | **Attribute Subcategory** | **Score** |
| --- | --- | --- |
| Composite (and Extreme Events) | Flooding | 100 |

**Table 33.** Environmental Attribute profile for the Commercial Property Owners beneficiary group.

| **Attribute Category** | **Attribute Subcategory** | **Score** |
| --- | --- | --- |
| Soil | Soil Quality | 10 |
|  | Substrate Quality | 10 |
| Water | Water Quality | 18 |
|  | Water Quantity | 17 |
|  | Water Movement | 17 |
| Composite (and Extreme Events) | Ecological Condition | 10 |
|  | Flooding | 18 |

**Transportation Beneficiaries**

**Table 34.** Environmental Attribute profile for the Transporters of Goods beneficiary group.

| **Attribute Category** | **Attribute Subcategory** | **Score** |
| --- | --- | --- |
| Composite (and Extreme Events) | Flooding | 100 |

**Table 35.** Environmental Attribute profile for the Transporters of People beneficiary group.

| **Attribute Category** | **Attribute Subcategory** | **Score** |
| --- | --- | --- |
| Composite (and Extreme Events) | Viewscapes | 5 |
|  | Flooding | 95 |

**Non-Use Beneficiaries**

**Table 36.** Environmental Attribute profile for the People Who Care beneficiary group.

| **Attribute Category** | **Attribute Subcategory** | **Score** |
| --- | --- | --- |
| Water | Water Quality | 5 |
| Fauna | Fauna Community | 5 |
|  | Edible Fauna | 5 |
|  | Keystone Fauna | 8 |
|  | Charismatic Fauna | 8 |
|  | Rare Fauna | 8 |
|  | Commercially Important Fauna | 5 |
|  | Spiritually / Culturally Important Fauna | 2 |
| Flora | Flora Community | 5 |
|  | Keystone Flora | 5 |
|  | Charismatic Flora | 2 |
|  | Rare Flora | 5 |
|  | Spiritually / Culturally Important Flora | 2 |
| Composite (and Extreme Events) | Sounds | 1 |
|  | Scents | 1 |
|  | Viewscapes | 8 |
|  | Phenomena (e.g. Sunsets, Northern Lights, etc) | 1 |
|  | Ecological Condition | 8 |
|  | Open Space | 8 |
|  | Flooding | 8 |

1. **Complete FEGS Scoping Tool output of priority scores**

**Table 37. BENEFICIARY GROUPS**: All beneficiaries that were identified as being a component of any one or more stakeholder groups in relation to the decision alternatives of the TRW site.

| **Beneficiary Category** | **Beneficiary Subcategory** | **Result** |
| --- | --- | --- |
| Non-Use | People Who Care | 21.55 |
| Transportation | Transporters of People | 10.09 |
| Learning | Students and Educators | 8.18 |
| Transportation | Transporters of Goods | 8.00 |
| Learning | Researchers | 7.96 |
| Recreational | Experiencers/Viewers | 5.17 |
| Governmental/Municipal/Residential | Public Sector Property Owners | 4.67 |
| Recreational | Hunters | 4.15 |
| Commercial/Industrial | Commercial Property Owners | 3.42 |
| Government/Municipal/Residential | Residential Property Owners | 3.39 |
| Subsistence | Water Subsisters | 3.11 |
| Recreational | Anglers | 3.02 |
| Agricultural | Livestock Grazers | 1.80 |
| Agricultural | Foresters | 1.79 |
| Commercial/Industrial | Timber/Fiber/Ornamental Extractors | 1.79 |
| Agricultural | Farmers | 1.63 |
| Recreational | Food Pickers/Gatherers | 1.47 |
| Agricultural | Aquaculturalists | 1.32 |
| Recreationl | Boaters | 1.14 |
| Inspirational | Spiritual and Ceremonial Participants | 1.01 |
| Inspirational | Artists | 0.84 |

**Table 38. ENVIRONMENTAL ATTRIBUTES:** All environmental attributes that were identified as being a component of any one or more beneficiary groups in relation to the decision alternatives of the TRW site.

| **Attribute Category** | **Attribute Subcategory** | **Result** |
| --- | --- | --- |
| Composite (and Extreme Events) | Flooding | 30.75 |
| Fauna | Edible Fauna | 7.89 |
| Composite (and Extreme Events) | Ecological Condition | 7.35 |
| Water | Water Quality | 5.53 |
| Composite (and Extreme Events) | Viewscapes | 4.70 |
| Composite (and Extreme Events) | Open Space | 4.27 |
| Fauna | Fauna Community | 4.08 |
| Water | Water Quantity | 3.55 |
| Flora | Flora Community | 3.47 |
| Fauna | Rare Fauna | 2.75 |
| Fauna | Keystone Fauna | 2.64 |
| Water | Water Movement | 2.63 |
| Fauna | Charismatic Fauna | 2.48 |
| Composite (and Extreme Events) | Earthquakes | 2.21 |
| Soil | Rare Flora | 1.77 |
| Soil | Keystone Flora | 1.71 |
| Fauna | Soil Quality | 1.30 |
| Composite (and Extreme Events) | Substrate Quality | 1.28 |
| Fauna | Commercially Important Fauna | 1.13 |
| Composite (and Extreme Events) | Sounds | 1.06 |
| Composite (and Extreme Events) | Phenomena | 0.89 |
| Composite (and Extreme Events) | Scents | 0.77 |
| Fauna | Spiritually/Culturally Important Fauna | 0.64 |
| Flora | Spiritually/Culturally Important Flora | 0.64 |
| Flora | Charismatic Flora | 0.58 |
| Fungi | Fungal Community | 0.48 |
| Atmosphere | Air Quality | 0.46 |
| Soil | Substrate Quantity | 0.32 |
| Fungi | Medicinal Fungi | 0.28 |
| Flora | Medicinal Flora | 0.27 |
| Composite (and Extreme Events) | Wildfire | 0.26 |
| Composite (and Extreme Events) | Extreme Weather Events | 0.26 |
| Atmosphere | Wind Strength/Speed | 0.24 |
| Flora | Edible Flora | 0.23 |
| Fungi | Edible Fungi | 0.23 |
| Other Natural Components | Presence of Other Natural Material for Artistic Use or Consumption | 0.22 |
| Fungi | Spiritually/Culturally Important Fungi | 0.19 |
| Other Natural Components | Fiber Material Quality | 0.17 |
| Other Natural Components | Fiber Material Quantity | 0.17 |
| Atmosphere | Sunlight | 0.06 |
| Atmosphere | Rare Fungi | 0.05 |
| Soil | Medicinal Fauna | 0.01 |
| Other Natural Components | Mineral/Chemical Quality | 0.01 |
| Other Natural Components | Mineral/Chemical Quantity | 0.01 |
